# Cellulose-Based Intelligent Responsive Materials: A Review

**DOI:** 10.3390/polym15193905

**Published:** 2023-09-27

**Authors:** Sisi Chang, Zhangzhao Weng, Chunmei Zhang, Shaohua Jiang, Gaigai Duan

**Affiliations:** 1 Jiangsu Co-Innovation Center of Efficient Processing and Utilization of Forest Resources, International Innovation Center for Forest Chemicals and Materials, College of Materials Science and Engineering, Nanjing Forestry University, Nanjing 210037, China; sisi_chang_lab@126.com; 2 Strait Institute of Flexible Electronics (SIFE, Future Technologies), Fujian Normal University, Fuzhou 350117, China; 3 Institute of Materials Science and Devices, School of Materials Science and Engineering, Suzhou University of Science and Technology, Suzhou 215009, China; cmzhang@usts.edu.cn

**Keywords:** cellulose, actuator, response, temperature, biomimetic robots

## Abstract

Due to the rapid development of intelligent technology and the pursuit of green environmental protection, responsive materials with single response and actuation can no longer meet the requirements of modern technology for intelligence, diversification, and environmental friendliness. Therefore, intelligent responsive materials have received much attention. In recent years, with the development of new materials and technologies, cellulose materials have become increasingly used as responsive materials due to their advantages of sustainability and renewability. This review summarizes the relevant research on cellulose-based intelligent responsive materials in recent years. According to the stimuli responses, they are divided into temperature-, light-, electrical-, magnetic-, and humidity-responsive types. The response mechanism, application status, and development trend of cellulose-based intelligent responsive materials are summarized. Finally, the future perspectives on the preparation and applications of cellulose-based intelligent responsive materials are presented for future research directions.

## 1. Introduction

At present, the rapid development of materials science and engineering, civil and structural construction engineering, and the manufacturing industry has greatly promoted the transformation of electronic technology from traditional rigid rectangular buildings to flexible, electronic, and various flexible building forms. Intelligent driving materials can convert external energy from external stimulus [1] into mechanical energy and bring shape changes [2]. It is widely used in soft robots [3], artificial muscles [4], actuators [5], and other fields. An actuator is a device that can convert universal input energy into mechanical motion. This broad definition has led to a wide variety of actuators with different classifications. According to the definition proposed by Constantinos et al. [6] in 1999, traditional actuators include electric, hydraulic, and pneumatic types. These actuators can utilize electromagnetic energy to provide rotary or linear motion (known since Faraday’s experiments in 1821 [7]). In the initial phase, soft actuators are mainly based on block and plate structures exhibiting in-plane deformation in the presence of variations in volume, alignment, and permutation. Subsequently, a great deal of research has focused on soft actuators with dual wafer or gradient structures, which involve reversible deformation between two-dimensional (2D) shapes and three-dimensional (3D) shapes. However, in this case, a limited number of deformation modes can be used, such as bending and twisting [8].

In nature, many biological phenomena utilize the principle of actuators, such as sunflowers growing toward the sunlight; the leaves of plants converting light energy into organic matter through photosynthesis; and pine cones falling from trees, contracting when exposed to water. In nature, many organisms use one-dimensionally shaped structures to adapt or change their living environment. Typically, animals, especially mammals, execute multiple bodily movements by controlling the contraction or relaxation of muscles attached to bones or other organs; sunflower stems exhibit phototropic bending toward sunlight to harvest more energy. These natural phenomena have inspired the development of novel soft actuators. Inspired by intelligent systems in the natural environment, people are committed to converting external stimulus such as electricity, light, heat, humidity, or magnetism into energy. Electric response actuators have advantages in energy conversion efficiency, the amount of stored energy, and controllability [9]. Optical responsive actuators are an intangible and remote control method with advantages in energy, wavelength selectivity, and ecological friendliness [10]. In this type of actuation, light is absorbed and converted into thermal or electrical energy, achieving photothermal and optoelectronic actuation [11]. Thermal and humidity responsive actuation typically relies on the expansion or contraction of materials caused by changes in temperature or humidity [12,13,14]. In addition, by assembling multi-layer materials with different responsibilities, their performance can be further improved or new properties can be added. For the magnetic-responsive actuation, magnetic fillers are added to soft materials to generate shape-changing forces following changes in external magnetic fields [15]. As single actuators, thermal-, optical-, electrical-, and magnetic-responsive actuators can only convert one type of energy into another. However, recently, single actuators have been unable to meet human needs, and more scientists have begun to study multi-responsive actuators [16], which has become a development trend.

As a crucial component of any machine, the primary purpose of an actuator [17] is to transform an input energy or signal into another form of energy or signal, which is subsequently released. Typically constructed from multiple layers of thin films, an actuator utilizes the asymmetric deformations from each layer as the driving force for its operation, making it highly responsive to changes from external stimulus [18]. With their vast potential for use in artificial muscles [19], neuroprosthetics bionics [20], precise control [21], and smart homes [22], it is crucial to explore alternative materials to petroleum-based actuators since they are non-degradable and non-renewable, which is contrary to the principles of sustainable development.

As the most abundant natural polymer in the world, cellulose is a sustainable and eco-friendly material that is increasingly being utilized as an actuator material. Cellulose is a macromolecular polysaccharide composed of glucose, which is the main component of plant cell walls [23,24]. It is a sustainable and renewable raw material with characteristics such as reusability, non-toxicity, environmental friendliness, biocompatibility, and biodegradability. It is one of the most abundant and commonly used biopolymers on Earth. Cellulose macromolecules are composed of a repetitive β-D-glucose unit, which is covalently linked by glycosidic bonds between hydroxyl groups [25], forming a linear macromolecule. Cellulose has strong hydrogen bond and high crystallinity within and between molecules, thus having excellent mechanical properties. The polymer chains of cellulose in natural cellulose fibers form a layered network structure through intermolecular and intramolecular bonds that are created by the abundant hydroxyl groups in the cellulose macromolecules. This particular structure also grants cellulose fibers exceptional moisture absorption and swelling properties, leading their responses to water molecules [26].

**Figure 1:**
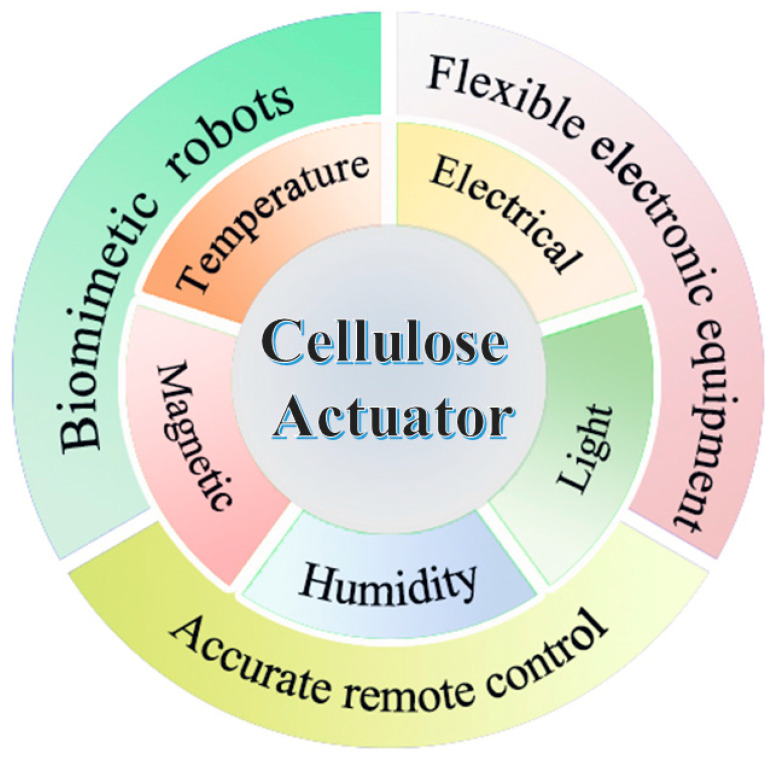
Response styles and applications of cellulose-based actuators.

So far, cellulose has been discovered as an intelligent material that can be used as sensors [27], actuators [28], smart devices [29], and so on. It can be used as Electroactive Paper (EAPap) [30], which has advantages such as light weight, flexibility, dryness, biodegradability, easy chemical modification, and cheapness [31]. Cellulose has become a green and renewable chemical resource, making great contributions to human development [32]. Figure 1 summarized the response styles and different applications of cellulose-based actuators.

## 2. Preparation of Cellulose-Based Responsive Materials

The structure of cellulose-based actuator polymers can be roughly divided into isotropic [33] and anisotropic structures [34]. Current methods to achieve complex deformation can be divided into two main categories. One is the non-homogeneous excitation of isotropic cellulose-based actuators or hydrogels, such as localized electric fields or localized light. The main principle is that the local stimulation for the complete cellulose-based actuator will only present locally uniform changes; however, multiple different degrees of stimulation will make the overall actuator uneven deformation. Through a reasonable structural design or program design, the complex deformation of the actuator can be realized.

The second is the anisotropic cellulose-based actuator, which can be used as the actuator layer therein by introducing the orientation structures or orientation patterns of the cellulose fibers. When the actuator is uniformly stimulated, the anisotropic structure causes the cellulose-based actuator to be subjected to different variations in each direction, generating forces of different strengths, which in turn cause the actuator to undergo complex deformation. Here we focus on the preparation method of anisotropic actuators that has been extensively studied. The anisotropic structures of cellulose-based actuators are mainly categorized into single-layer (also called gradient), bilayer, and multilayer structures.

### 2.1. Single-Layer Structure

**Figure 2:**
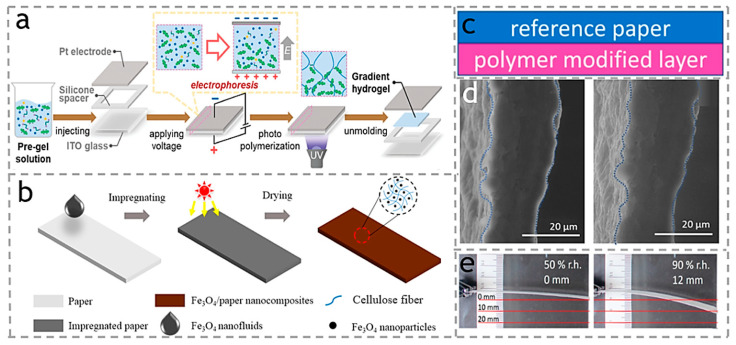
(**a**) A single gradient hydrogel actuator utilizing cellulose nanocrystals (CNCs). Reproduced with permission [35]. Copyright 2023. The Royal Society of Chemistry. (**b**) Fabrication process of Fe_3_O_4_/paper nanocomposite via a low-cost blending method. Reproduced with permission [37]. Copyright 2018. ELSEVIER. (**c**) Single-layer actuator consisting of polymer-modified paper. (**d**) Cross-sectional ESEM images of single-layer films at different RH. (**e**) polymer modified “layer” pointing down, in the 50% RH and 90% RH. Reproduced with permission [36]. Copyright 2023. Biomimetics.

An anisotropic monolayer actuator is an ideal material, as it eliminates the necessity for interface bonding or complexities in the production process commonly associated with multi-layer structures. Both the gradient distribution of some fillers and the gradient distribution of polymer chains can produce shape changes in cellulose-based actuators with gradient structures. Therefore, the ways to prepare cellulose-based actuators with a gradient structure can be broadly classified into two categories. (1) The construction of gradient structures is realized by generating an asymmetric distribution of polymer chains in vivo. For example, Roop sung et al. [35] employed electrophoresis to manufacture a single gradient hydrogel actuator utilizing cellulose nanocrystals (CNCs). As shown in Figure 2a, this hydrogel actuator showed temperature-responsive bending behavior, whereby the bending angle progressively increased over time, resulting in the formation of a semicircular shape, as the temperature rose from 25 °C to 50 °C within four minutes. As shown in Figure 2c, Schäfer et al. [36] developed a single-layer actuator consisting of polymer-modified cellulose paper, which shows a bending response when exposed to moisture, as shown in Figure 2d. The researchers showed that this design not only minimizes the likelihood of delamination, but also simplifies the overall design and construction processes in contrast to multilayer structures. (2) The second category is embedding stimuli-responsive nanoparticles in the polymerization process and utilizing their migration in an applied electric or magnetic field to form a gradient arrangement. For example, Wang et al. [37] immersed Fe_3_O_4_ nanoparticles into nitrocellulose fibers of chromatography paper. As shown in Figure 2b, they observed that the end of the fiber showed augmented deflection and bending angle as the magnetic field strength increased, indicating the reversibility of this effect. Additionally, the fiber exhibited the ability to pick up paper masses twice its own weight under magnetic stimulation.

Fibrous cellulose nanofibers (CNFs) possess a high aspect ratio, which facilitates the intercalation of water molecules within the cellulose network and enhances actuation performance. Lopes et al. [38] prepared CNF monolayer membrane actuators with the carboxymethylation of CNF. As shown in Figure 2d,e, upon observation of the film under 100% relative humidity, a substantial volume expansion was observed through microscopic morphology analysis.

### 2.2. Double-Layer/Multi-Layer Structure

At the present stage, the preparation of bilayer actuators mainly consists of two methods: layer-by-layer deposition [39] and reversible switch assembly [40]. In the preparation process of layer-by-layer deposition, the polymer of the second layer will penetrate into the first layer in a small amount, forming an interpenetrating network at the interface and thus making the two layers closely connected. For example, Liu et al. [41] electrospun PNIPAM and TPU into a bilayer-structured actuator, where the interfaces can be strengthened by the hot-pressed melting TPU, and the thermal responsiveness of the actuator can be modulated by adjusting the thickness of the two fiber membranes. For the reversible switch assembly method, generally the actuator layers are connected by host–guest interactions or hydrogen bonding, which makes the layers tightly connected together to form a bilayer structure actuator. For example, Ma et al. [42] constructed a bilayer-structured hydrogel actuator by a supramolecular building block assembly method, in which they prepared a responsive body hydrogel with a β-cyclodextrin structure and carboxyl groups and a non-responsive guest hydrogel containing ferrocene groups. After that they utilized such reversible host–guest interactions between β-cyclodextrin and ferrocene to assemble a hydrogel actuator that could bend with the changes of pH value and ionic strength.

**Figure 3:**
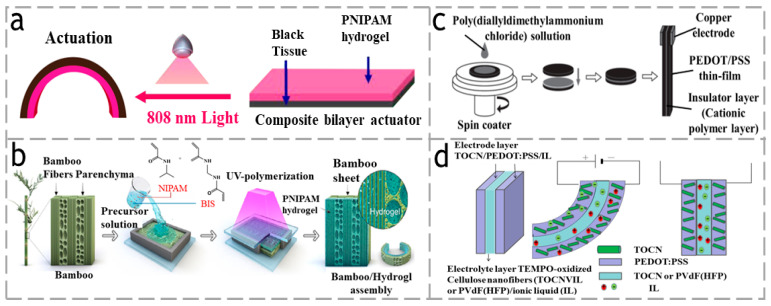
(**a**) A paper/PNIPAM composite hydrogel actuator. Reproduced with permission [43]. Copyright 2022. Polymers. (**b**) A bamboo/PNIPAM hydrogel composite actuator. Reproduced with permission [44]. Copyright 2021, AMER CHEMICAL SOC. (**c**) A single-layer actuator consisting of polymer-modified paper. Reproduced with permission [46]. Copyright 2020. Japan Oil Chemists’ Society. (**d**) TOCN/PEDOT: PSS/IL electrode actuator. Reproduced with permission [47]. Copyright 2021. ELSEVIER.

In addition to the two methods mentioned above, many researchers have bonded hydrogels and polymers together by simple methods of preparing polymers, such as in in situ polymerization, which is one of the effective methods for synthesizing nanocomposites and appears to be particularly useful in the synthesis of composites based on monomers. As shown in Figure 3a, Wu et al. [43] prepared a paper/PNIPAM composite hydrogel actuator. Due to the in-situ polymerization of NIPAM monomer integrating the thin paper and hydrogel layers together, and the strong bonding of the bilayer structure, the hydrogel composite actuator can achieve a variety of controlled deformations, showing remarkable photothermal responsiveness. As shown in Figure 3b, Chen et al. [44] also used the in situ polymerization method by applying a N-isopropylacrylamide (NIPAM) monomer on the surface of a bamboo sheet to form a continuous layer, followed by UV polymerization to convert it into a PNIPAM hydrogel, resulting in an anisotropic bamboo/PNIPAM hydrogel complex (ABH) actuator. With the difference of adopting a morphogenetic approach based on a natural bio-template of bamboo, they demonstrated how its biomimetic structure can integrate the features of high-strength, fast response, and programmable composite deformation into a single device.

In general, the ability to respond to external stimuli of the single-layer cellulose-based actuator is limited. Hence, researchers are increasingly incorporating additional functional polymeric materials and anisotropic structural materials to enhance its performance [45]. For instance, the inclusion of conductive polymers can impart outstanding electroactive characteristics to the actuator. For example, as shown in Figure 3c, Wu et al. [46] successfully fabricated a three-layer cellulose paper actuator with a capacitor-type design. This actuator consists of two layers of doped poly (3-styrene sulfonate) (PEDOT/PSS) poly (4,4-vinylidene dithiophene) thin films, with another polyelectrolyte layer sandwiched in between. The actuator demonstrates a stable electromechanical deformation that is symmetric in both directions. This cellulose paper-based actuator, with a thickness of 48 μm, achieves a maximum displacement of 0.05 mm when operated at a voltage of 5.8 V and a frequency of 1.5 Hz. As shown in Figure 3d, Naohiro et al. [47] utilized a gel electrode composed of 2,2,6,6-tetramethylpiperidine-1-oxyl (TEMPO)-oxidized cellulose nanofibrils (TOCN), PEDOT:PSS, and an ionic liquid (IL) for the preparation of a novel actuator. This gel electrode actuator demonstrates exceptional electrical conductivity as well as ion conductivity properties.

## 3. Response Mechanism of Actuators

### 3.1. Temperature Response Mechanism

Temperature response refers to the transformation of a material’s state through changes in the external environmental temperature [48,49], from the interaction between polymer and water to the interaction between polymer and polymer. The heat source is usually heating, electrothermal, and photothermal [50,51].

### 3.2. Light Response Mechanism

Photoactuation is a wireless- and remote-control method that has advantages in energy, wavelength selectivity, and ecological friendliness. In this type of actuation, light is absorbed and converted into thermal, chemical, or electrical energy, achieving photothermal, photochemical, and optoelectronic actuation.

The photo-thermal-responsive actuator, with its unique dual photo and thermal responses, can achieve process control and is remotely driven by a special light source, thus realizing the comprehensive regulation of the actuator. It is worth noting that for the photothermal response, the required light generally refers to the near-infrared light or lasers, which possess high energy and non-radiation penetration. Thus, the light-controlled regulation of the actuator is of great significance in areas that are inconvenient for people to access.

### 3.3. Electrical Response Mechanism

Actuation by electricity is working based on the electric energy, which can be converts into torque, then drives the actuator. Because of the fast conductive and clean energy of electricity, the electrical response has the advantages of being quick, precise, and clean. Many previous studies have found that cellulose has piezoelectric properties [52]. The high crystallinity and abundance of polar hydroxyl groups make cellulose contain a large number of dipoles with strong electron-donating ability, and thus cellulose can be used to fabricate electrically responsive actuators. 

### 3.4. Magnetic Response Mechanism

Magnetic actuation is mainly from the addition of magnetic fillers to soft materials, which follow changes in the external magnetic field to produce changes in shape or torque. As is well known, the surface of cellulose contains abundant hydroxyl groups and the porosity of cellulose. Magnetic particles can be easily added with cellulose to produce composites. The commonly used ferromagnetic particles include Fe_3_O_4_ [53], Fe_2_O_3_ [54], MnFe_2_O_4_ [55], CoFe_2_O_4_ [56], and so on. When the external magnetic field changes, each magnetic particle will have a similar trend and undergo a relative adjustment. Due to this adjustment, the cellulose composites can elongate or contract, leading to the corresponding actuation.

### 3.5. Humidity Response Mechanism

It should be noted that cellulose contains a large number of hydrogen bonds, and the interaction of hydrogen bonds provides the polymer with excellent hygroscopicity. The absorption and desorption of water molecules by cellulose can significantly change its volume. At the same time, the abundant hydroxyl and carboxyl groups on the molecular chain of cellulose result in cellulose itself being a hygroscopic material. Humidity-responsive actuators have attracted great interest due to the presence of water in nature and the concept of green environmental protection. The design principle of humidity actuation [57] is that materials with different hydrophilicity have different responses to the humidity, and their materials will undergo varying degrees of deformation due to swelling. When external moisture or humidity increases, the materials will undergo a corresponding actuation. This material system has great application potential in multifunctional or intelligent actuators.

## 4. Cellulose-Based Temperature-Responsive Actuators

Temperature-responsive actuators can undergo a volumetric phase transition through the conversion of its own hydrophilicity. 

Thermal-responsive materials are qualified candidate materials that can be directly used as actuators by thermal stimulus [58]. Therefore, this kind of hydrogel generally has a lower critical solution temperature (LCST) or a upper critical temperature (UCST) [59], and the temperature-responsive hydrogel will expand or shrink when the temperature is higher or lower than the critical temperature. For example, poly(N-isopropylacrylamide) (PNIPAM), as a typical thermal-responsive polymer [60,61,62], has been widely studied by researchers. The LCST of PNIPAM is approximately 32 °C [63], and the hydrophilic/hydrophobic state of PNIPAM will change nearby. Since the thermal-responsive PNIPAM micro gel was reported in 1986, PNIPAM hydrogel has been frequently used as the raw material of actuators.

**Figure 4:**
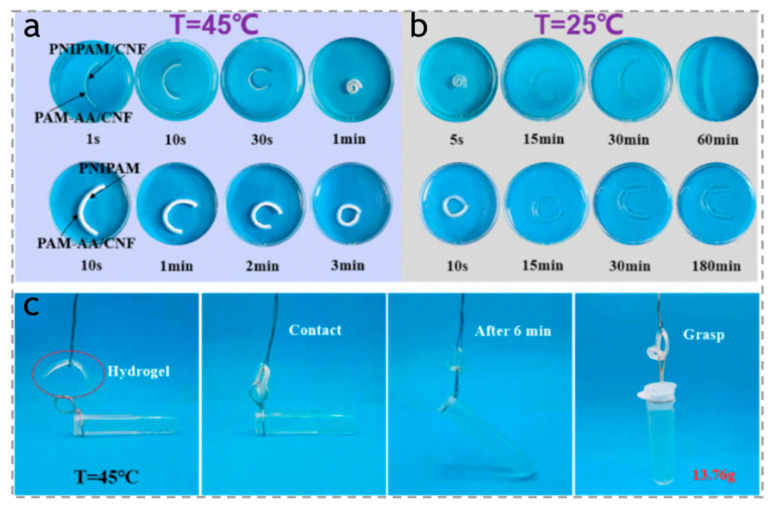
(**a**) Pictures showing the bending of PAM-AA/CNF + PNIPAM/CNF hydrogel strip and PAM-AA/CNF + PNIPAM hydrogel strip at 45 °C in water bath and (**b**) their unbending at 25 °C. (**c**) Demonstrations of bilayer hydrogel as temperature-controlled manipulators at 45 °C. Reproduced with permission [64]. Copyright 2022. ELSEVIER. The red circle indicates the bilayer hydrogel sample.

In general, the pure PNIPAM hydrogel is isotropic, and only has a simple volume contraction or expansion under the condition of temperature change. Many researchers focus on the cellulose-based composites with PNIPAM to achieve a complex deformation. For example, as shown in Figure 4a,b, Liu et al. [64] have successfully prepared a bilayer hydrogel actuator with a vertically oriented anisotropic structure, which exhibits temperature-responsive behavior. The top layer of the hydrogel is constructed using a double network of poly(N-isopropylacrylamide)/cellulose nanofiber (PNIPAM/CNF), while the bottom layer consists of a double network of polyacrylamide-co-acrylic acid/cellulose nanofiber (PAM-AA/CNF). This bilayer hydrogel actuator demonstrates remarkable driving capability and mechanical performance, as it is capable of bending at a high speed of 70.10 (°/s) and can lift a weight that is 21 times its own weight, as shown in Figure 4c.

**Figure 5:**
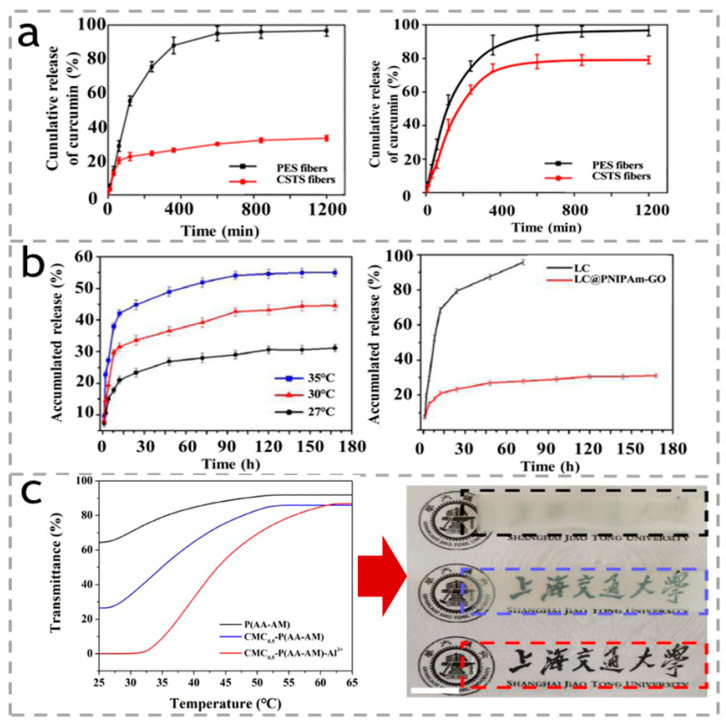
(**a**) Release profiles of curcumin from PES and CSTS ultrafine fibers at 20 °C and 60 °C; reproduced with permission [65]. Copyright 2019. ELSEVIER. (**b**) Effect of temperature on the release behavior of that, and the release behavior of free LC and LC@PNIPAm-GO at RT. Reproduced with permission [66]. Copyright 2021. ELSEVIER. (**c**) Temperature-transmittance curves of the P(AA-AA), CMC_0.5_-P(AA-AM), and CMC_0.5_-P(AA-AM)-Al^3+^ hydrogels (**left**) and the corresponding photographs of each hydrogel in a 25 °C water bath; the scale bar is 1 cm. Reproduced with permission [67]. Copyright 2020. American Chemical Society. The Chinese words indicated the name of Shanghai Jiaotong University.

In another work, Wei et al. [65] prepared a complex hydrogel based on dextran and PNIPAM. When the temperature is higher than 32 °C, the PNIPAM molecular chain will shrink, thereby extruding the curcumin loaded in the hydrogel (Figure 5a). As shown in Figure 5b, Wang et al. [66] prepared a multifunctional composite nanocarrier poly(N-isopropylacrylamide)-modified graphene oxide (PNIPAM-GO), and through a simple physical adsorption process, load λ- Cyhalothrin (LC) onto PNIPAM GO nanocomposite carriers. However, it is a pesticide formulation that can adjust the pesticide release based on changes in the external temperature and has a certain intelligent effect on crop growth. As shown in Figure 5c, Chen et al. [67] integrated conductive hydrogel (carboxymethyl cellulose) and thermal-responsive poly(N-isopropylacrylamide) (PNIPAM) hydrogel to form a double-layer hydrogel, which has the ability to respond to environmental temperature changes. More interesting is that because the hydrogel has different degrees of expansion and contraction under different thermal stimuli, it can produce two-way bending. The dotted lines in Figure 5c indicated the transmittance of the hydrogel reversibly changes when the temperature changes.

In addition, Joule heating is also a method of using electrical energy for heating, which involves using thermal conductive materials as an electric heating layer, such as carbon nanotubes [68,69], MXene [70,71], and so on. Conductive materials can be used as electric heating layers, and then compounded with natural or synthetic cellulose to form a double-layer actuator. For example, Wei et al. [72] reported a CNF (cellulose nanofiber)—MXene (Ti_3_C_2_T_x_) nanosheet-TA (tannic acid) composite, in which MXene nanosheets have excellent conductivity, resulting in good Joule thermal performance of composite thin-film actuators. 

**Figure 6:**
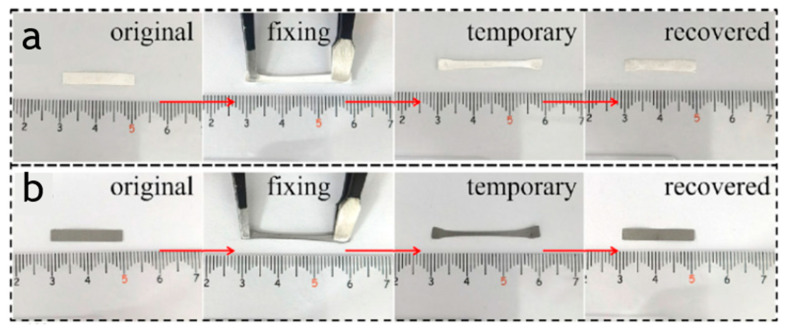
(**a**) Photothermal conversion performance of CA nanofoborous composites. (**b**) NIR light-induced shape memory behaviors of CAC. Reproduced with permission [73]. Copyright 2023. ELSEVIER.

Shape-memory polymer (SMP) can perform repeatable shape transformation under various stimulus; thus, it has great application prospects in the field of actuators and has been widely studied. As shown in Figure 6a, Bai et al. [73] prepared an electrospun cellulose acetate (CA)/carbon nanotube nanofiber composite (CAC) with both shape memory performance and self-powered sensing performance for the first time. The material was stretched to 90% strain at 50 °C (higher than its T_g_), cooled to room temperature to fix the temporary shape, and then reheated to 90 °C to restore its original shape. As shown in Figure 6b, it is clearly observed that CAC has the ability to fully restore its original shape, while also having a shape fixation and recovery rate of over 95%. They also demonstrated that the shape memory properties of the material are not related to the manufacturing method and carbon nanotube content, but only to the CA itself.

## 5. Cellulose-Based Light-Responsive Actuators

Light-responsive actuators using light as a stimulus source can not only be process-controlled, but can also be remotely driven by a special light source, thus realizing the comprehensive regulation of the actuator. It is worth noting that, in some inconvenient entering areas, the intelligent light-controlled regulation of the actuator is very meaningful.

**Figure 7:**
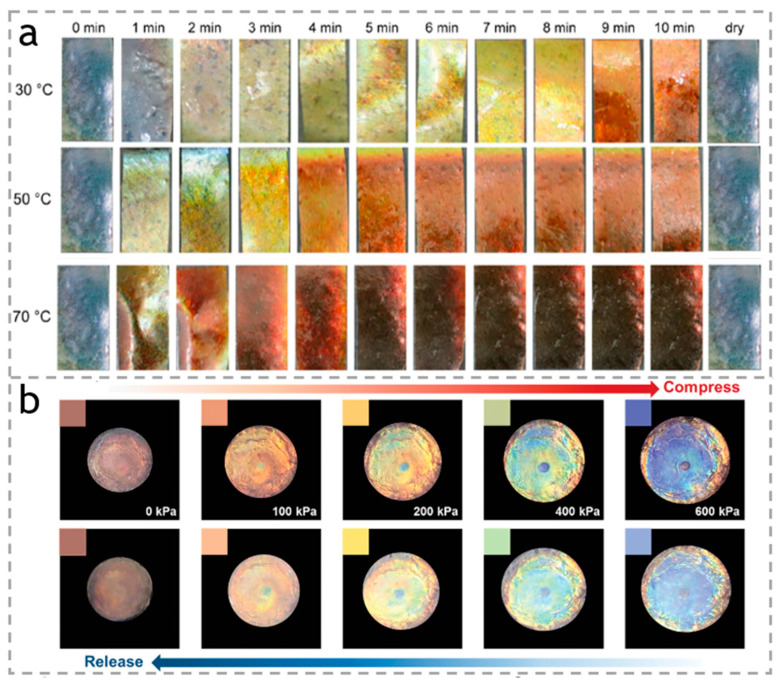
(**a**) Images of photonic film immersed in DMSO at various temperatures. Reproduced with permission [76]. Copyright 2023. Wiley-VCH Verlag. (**b**) Digital photos of the sample during compressing and releasing process. Reproduced with permission [77]. Copyright 2019. SPRINGER.

Many plants around the world have anisotropic cellulose structures, which play an important role in determining the different forms and colors of plants. Color variations in plants are mainly attributed to pigments and dye molecules. In addition to the structural color of the plant itself, pigments in plants can undergo color changes through selective absorption of light by their internal structures as well as scattering and refraction of light [74]. Cellulose nanocrystals (CNC) have emerged as prime candidates for manufacturing materials with unique optical characteristics [75], owing to their high aspect ratio and intermolecular interactions. Sun et al. [76] combined cellulose nanocrystals (CNCs) with different surface charge density and particle size with phenol glutaraldehyde resin and graphite oxide in a certain proportion to prepare composite materials of different colors, as shown in Figure 7a. After immersion in water for 25 s and in dimethyl sulfoxide (DMSO) for 20 min, the color of the films changed from green to red, but even after immersion for 3 months, other solutions with smaller polarity would not change color, indicating that it has a good degree of stability. This is a straightforward color change that only takes place when the substance is immersed in a solution. Inspired by the color-changing mechanism observed in neon lights, Li et al. [77] created a bionic smart hydrogel using cellulose nanocrystals. This hydrogel demonstrates a dual response to both pressure and temperature. As shown in Figure 7b, as vertical pressure is applied, the hydrogel’s color changes from red to orange, yellow, green, and blue. Moreover, by maintaining a constant external pressure and varying the temperature, the hydrogel is capable of undergoing a comparable rainbow-like color transformation. Even more remarkable, Orelma et al. [78] developed optical cellulose fibers by coating regenerated cellulose long filaments with acetic acid fibers. These fibers possess mechanical properties that are comparable to textile fibers. However, when light is observed in the range of 500–1400 nm, the cellulose fiber exhibits an attenuation constant of 3.1300 dB/cm at 6 nm. When the fiber is immersed in water, a substantial reduction in light intensity is achieved, indicating the potential use of this optical cellulose fiber as water sensors.

**Figure 8:**
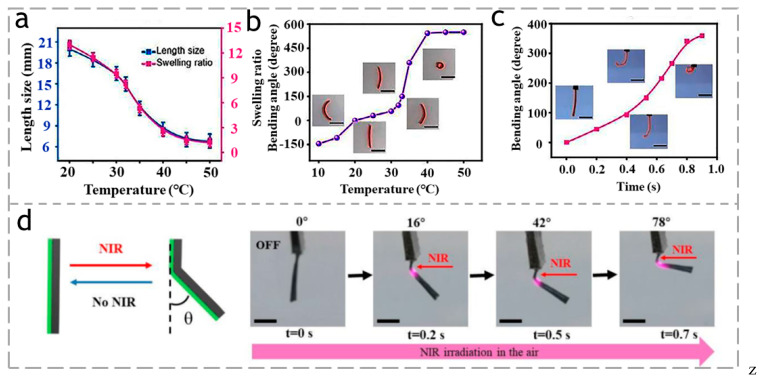
(**a**) The equilibrium swelling rate and length size of PNIPAM hydrogel at different temperatures. (**b**) The bending equilibrium angle of the wood-PNIPAM composite hydrogel actuator at different temperatures. (**c**) Curve of bending angle over time of the wood-PNIPAM composite hydrogel actuator in 40 °C warm water. Reproduced with permission [84]. Copyright 2022. ELSEVIER. (**d**) The illustration of folding angle for bamboo/PNIPAM composite hydrogel actuator and the optical photos of bamboo/PNIPAM composite hydrogel actuator locally irradiated by NIR light. Reproduced with permission [85]. Copyright 2022. ELSEVIER.

In addition to the single temperature conversion mentioned above, photothermal conversion is another actuation mechanism. The photothermal response actuator can convert light energy into heat from carbon nanotubes [79], carbon nanorods [80], graphite oxide [81], and other materials. Due to the high photothermal conversion and absorbance of near-infrared (NIR) energy, the near-infrared laser is usually used as the light source. For example, Zhao et al. [82] prepared a new type of double-layered nano-composite hydrogel actuator containing nano fibrillar cellulose (NFC), poly (N-isopropylacrylamide)-clay, and GO. GO absorbed near-infrared laser in the hydrogel and converted it into energy leading to a temperature rise, thus making the volume of the composite hydrogel actuator shrink and bend. Li et al. [83] self-assembled cellulose nanocrystals (CNCs), thin films, and polyurethane (PU) substrates, as well as dispersed in situ synthesized silver nanoparticles (AgNPs) into the PU matrix. Due to the high photothermal conversion efficiency and thermal conductivity of AgNPs, the elastomer exhibits excellent photothermal conversion efficiency and thermal conductivity. As shown in Figure 8, Chen et al. [84,85] made wood/PNIPAM and bamboo/PNIPAM composite hydrogel actuators, respectively, by combining wood and bamboo with hydrogel through simple and rapid in situ polymerization. The natural anisotropy of wood and bamboo [86,87,88,89] shows different shapes of deformation under near-infrared light and can be remotely controlled by light response actuation.

## 6. Cellulose-Based Electrically Responsive Actuators

**Figure 9:**
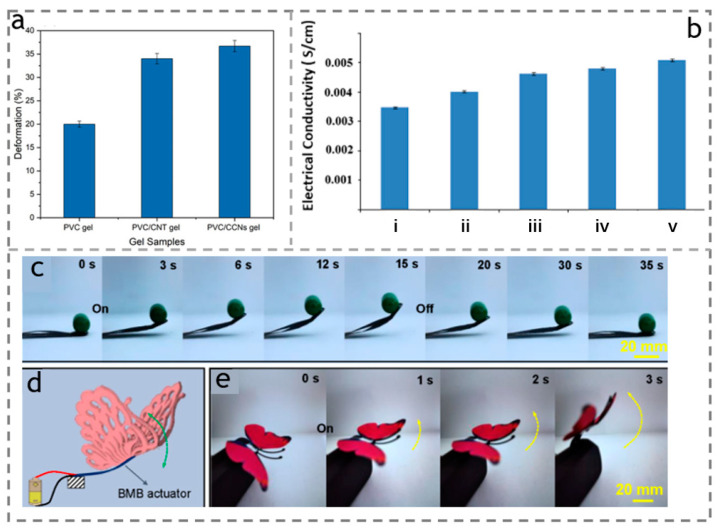
(**a**) Deformation of the pure PVC gel, PVC/CNT, and PVC/CCNs composite gel. Reproduced with permission [91]. Copyright 2023. AMER CHEMICAL SOC. (**b**) Effect of Gr loading on electrical conductivity of samples (i) MCC, (ii) MCC–BMCl, (iii) MCC–BMCl–0.1Gr, (iv) MCC–BMCl–0.25Gr, (v) MCC–BMCl–0.5Gr film. Reproduced with permission [95]. Copyright 2015. ELSEVIER. (**c**) Optical images showing the BMB actuator lifting up a 200 mg weight under an applied voltage of 4 V. (**d**) Schematic illustration showing the structure of the moveable butterfly. (**e**) Optical images of the butterfly during upward movement (driving voltage: 4 V). Reproduced with permission [96]. Copyright 2022. American Chemical Society.

Intelligent electrically responsive actuators have the advantage of simple operation and excellent controllability. These soft actuators can either convert electrical energy directly into mechanical energy or drive thermally responsive materials through Joule heating. Previously, it was reported that cellulose-based electroactive paper (EApap) [30] is used as an electric-responsive actuator. This type of actuator is made of a piece of cellulose paper, and its surface is composed of two electrodes. In fact, these two electrodes are basically used to determine the performance of cellulose paper [90]. The cellulose of the paper is arranged through the regeneration process, and usually the solid material undergoes Coulomb interaction at higher voltages, leading to bending and responses. Muhammad et al. [91] prepared a new type of composite gel from functionalized cellulose carboxylate nanocrystals (CCNs) and polyvinyl chloride (PVC). Under the excitation of the specified DC voltage (1000 V), the deformation of the actuator was about 36.7%, as shown in Figure 9a.

In addition to the direct conversion mentioned above, cellulose can also be combined with some conductive materials to form a composite film, achieving more efficient electroactive properties. For example, MXene [92,93] and graphene nanosheet (GN) [94] have excellent conductivity and chemical stability, reducing the Joule loss during conversion into electrical energy. Sen et al. [95] demonstrated that loading graphene nanosheets onto a mixture of microcrystalline cellulose powder (MCC), 1-butyl-3-methylimidazolium chloride (BMCl), and N,N-dimethylacetamide (DMA) results in better electroactive performance of the cellulose-based composite actuator, as shown in Figure 9b. Tang et al. [96] prepared a three-layer composite membrane as flexible actuator (called BMB). They sandwiched pure MXene membrane between polypropylene (BOPP) and bacterial cellulose (BC) after wet expansion and biaxial stretching. The BMB actuator could quickly lift a weight of 200 mg under a voltage of 4 V, as shown in Figure 9c, and gradually returned to its original state after the voltage was turned off. They also used tape to stick a pair of plastic butterfly wings (approximately 435 mg) to the free end of the RMB actuator. When the input voltage is turned off, the butterfly wings will sag due to their own weight. However, when the voltage of 4 V is turned on, the actuator quickly lifts the butterfly wings, and the work conducted when lifting is higher than most actuators, as shown in Figure 9d,e.

Conductive polymers (CPs) can also be used as another component of actuator systems [97]. Due to their low price, affordability, high conductivity doping state, and large storage capacity, they are very suitable for making electrochemical capacitors. Among them, poly (3,4-ethylenedioxythiophene) (PEDOT) is considered as a very useful CP due to its conductivity, stability, and processability [98]. In order to investigate the actuation mechanism of electric-responsive actuators, Chalil et al. [99] prepared a poly (3,4-ethylenedioxythiophene) polystyrene sulfonate (PEDOT:PSS)/bacterial cellulose bilayer actuator. The electric response was performed using HCL, KCl, and LiCl electrolytes. They found that the actuator was related to the hydrodynamic size of the cations presented in the electrolyte. The experimental results also indicate that the PEDOT: PSS/bacterial cellulose bilayer actuator has a cation-dominated driving mechanism. Driven by the external voltage, the internal ions will move in the active layer, resulting in interaction, cross-linking between groups, etc., finally leading to the change of material volume and deformation actuation. Therefore, many researchers prepare ionic conductive hydrogels or actuators through ionic liquids. Naohiro et al. [100] prepared a novel actuator with a cellulose nanofiber/poly (3,4-ethylenedioxythiophene): poly (4-styrene sulfonate)/ionic liquid (CNF/PEDOT: PSS/IL), which replaces carbon nanotubes (CNTs) with a CNF skeleton. The maximum strain and maximum generated stress exhibited simultaneously exceeded the strain and maximum stress by PEDOT: PSS/IL actuators without cellulose nanofibers (CNFs), indicating that cellulose-based materials exhibit better strain performance in actuator devices.

**Figure 10:**
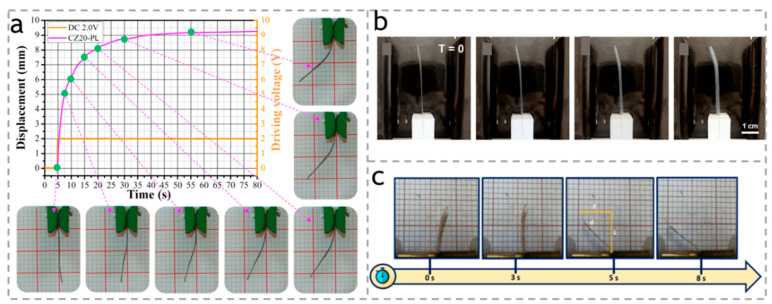
(**a**) Electromechanically deformed shapes of the CZ-PL muscular actuator under a step excitation of 2.0 V. Reproduced with permission [102]. Copyright 2017. ELSEVIER. (**b**) Swelling and disintegration of a pure CNC hydrogel cantilever during actuation testing in 3 mm NaCl electrolyte at T = 0 and after 1 min and 2 min at 10 V, and subsequently 1 min after switching voltage to −10 V. Reproduced with permission [103]. Copyright 2021. AMER CHEMICAL SOC. (**c**) Bending response as a function of time for NaCMC/[Ch][DHP] composite at a 100 mHz frequency and 8 V. Reproduced with permission [104]. Copyright 2020. MDPI.

In another report, Wang et al. [101] prepared a low-pressure ion actuator based on sulfonated cellulose nanocrystal (SCN), microfibrillated cellulose (MFC), graphene nanoplatelets (GN), and ionic liquid (IL, [EMIM][BF4]). This actuator exhibited a significant peak displacement of 0.1 mm at 1 V and 6.6 Hz. Furthermore, their team developed a high-fidelity bioelectronic muscle actuator based on a carboxymethyl cellulose bacterial cellulose (CBC) membrane using a simple zinc oxide (ZnO) particle leaching (PL) method. The membrane showed a remarkable improvement of 70.63%, 22.50%, and 18.2% in ionic liquid absorption capacity, ion exchange capacity, and ionic conductivity of CBC, respectively. As shown in Figure 10a, the bending deformation of the CZ-PL membrane-based actuator was 5.8 times larger compared to the pure CBC-based actuator [102].

Lai et al. [103] proposed pure cellulose nanocrystalline films as biomass actuators in ionic solutions. They expanded the CNC film in 0.1 M NaCl and cut it into rectangular cantilevers, which were installed in a simple dual motor test cell. A 10 V field was applied to NaCl electrolyte solutions of different intensities. The CNC cantilevers showed reversible driving with an alternating electric field potential for up to 20 min without mechanical failure; then, they compounded CNC with polyacrylamide. As shown in Figure 10b, this nanocomposite hydrogel showed reversible actuation at 1.4 °/s. Under 10 V, there was no mechanical failure during 60 min of experimental operation. At the same time, they also made a comparison. The sample without CNC did not show obvious actuation, which further confirmed that CNC had an electric-responsive actuation behavior. As shown in Figure 10c, Correia et al. [104] prepared cellulose nanocrystals with different surface charges (neutral, positive, and negative) and incorporated an increasing amount of IL 2-hydroxyethyl trimethylammonium dihydrogen phosphate ([Ch] [DHP]) (10 and 25 wt%) into the CNC master matrix. Regardless of the surface charge of the CNC, an increase in conductivity was observed when IL was incorporated into the CNC matrix. When a voltage of 100 V was applied at a frequency of 9 mHz, a maximum bending displacement of 8 mm was obtained.

## 7. Cellulose-Based Magnetic-Responsive Actuators

Intelligent magnetically responsive soft actuators for bio-inspired soft actuators offer unique advantages over other soft actuators. These advantages include: (1) non-contact remote control that can penetrate a wide range of materials; (2) precise manipulation by controlling the direction and strength of the magnetic field; (3) enhanced controllability by independently varying the magnetic field and gradient; (4) the ability to generate a magnetic alternating current (AC) for use in applications such as magnetothermal therapy; and (5) scalability and controllability of the actuator, which ranges from the nanoscale to the macroscopic level. Their disadvantage is that they require complex and bulky external devices to precisely control the magnetic field and its gradient.

**Figure 11:**
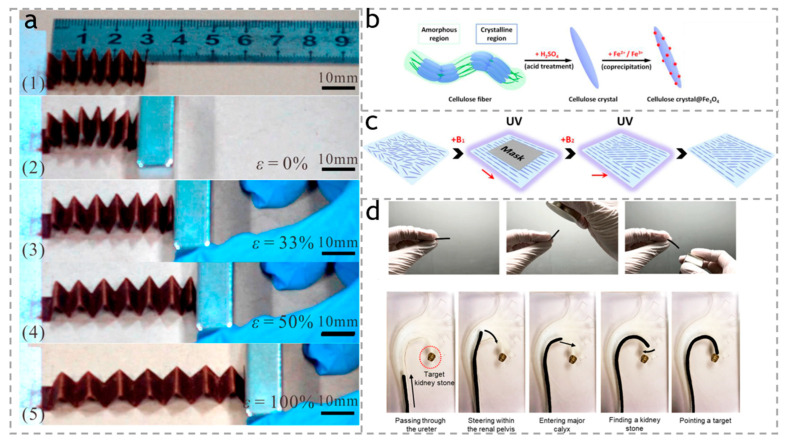
(**a**) Performance of accordion-shaped Fe_3_O_4_/paper actuator subjected to tunable magnetic field. (1–5) show actuator deformation magnetically actuated at specific normal strains. Reproduced with permission [37]. Copyright 2018. ELSEVIER. (**b**) Digital images of cellulose microcrystals containing Fe_3_O_4_ nanoparticles. (**c**) Scheme showing the lithography process for patterning a polymer film that contains magnetic cellulose microcrystals with different orientations in different regions. Reproduced with permission [105]. Copyright 2020. ELSEVIER. (**d**) Steering of the nanocellulose actuator string. Reproduced with permission [107]. Copyright 2021. American Chemical Society.

As shown in Figure 11a, Wang et al. [37] made a cellulose paper/Fe_3_O_4_ nanocomposite actuator through a low-cost blending. The actuator is driven by a magnet with a magnetic strength of about 230 mT, and the maximum deformation and strain achieved by the actuator are 30 mm and 100%, respectively.

Cellulose can be modified with magnetic materials, making it have excellent performance in the magnetic field. As shown in Figure 11b, Chen et al. [105] developed a nanocomposite, which is composed of rod-like cellulose microcrystals with magnetite nanoparticles attached to its surface. This design takes advantage of the optical transparency and birefringence of cellulose microcrystals and their anisotropic shape. When their surfaces are modified by magnetite nanoparticles, their anisotropic shape enables them to effectively align. Only a small amount of magnetite nanoparticles is needed to conduct such a real-time and reversible orientation control of cellulose microcrystals, which thus ensuring the high transparency of the system, as shown in Figure 11c.

Tomás et al. [106] produced a hybrid of cellulose nanocrystals decorated with magnetic nanoparticles. This actuator can synergistically promote hASC tension through mechanical sensing mechanisms and may regulate its paracrine signal transduction that promotes healing, thereby jointly promoting the improvement of the regenerative potential of engineered tendon grafts. Kim et al. [107] synthesized a dense surface layer of cellulose nanofibers (made from Acetobacter xylinum), enclosing magnetic nanoparticles (MNPs) in a solid matrix to form an actuator string with response to external magnetic fields. The nanofiber actuator string is convertible in order to adapt to various shapes of tubular structures on the cross-section, reducing friction and stress on organ tissue walls due to its softness and plastic deformation. As shown in Figure 11d, such nanofiber skin strings can be bent at acute angles through magnetic actuation and can be used as endoscopic guides to reach targets deep in the kidney model.

## 8. Cellulose-Based Humidity-Responsive Actuators

Intelligent humidity-responsive actuators can be used in the stimulus of liquid (moisture) or vapor (humidity). Moisture gradients and changes in humidity are common phenomena in nature. Various types of plants (e.g., mimosa and pinecones) respond to water stimuli. These water-responsive plants (also known as tidal grasses) attribute water-responsiveness and shape-altering properties to their bilayer structure, with each layer responding differently to water stimuli, resulting in anisotropic deformation of the bilayer structure.

**Figure 12:**
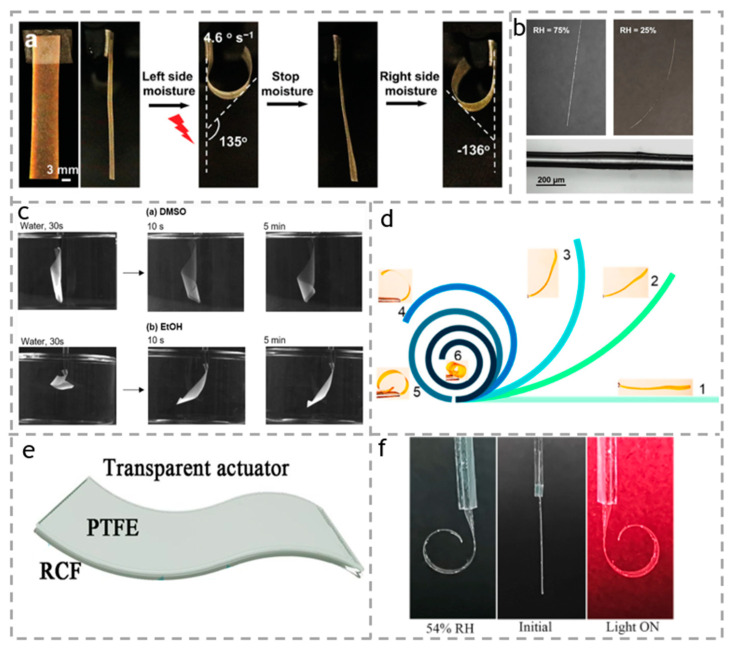
(**a**) A red rectangular CNC-PP film could rapidly carry out bidirectional moisture actuation at a speed of 4.6° s^−1^. Reproduced with permission [108]. Copyright 2022. WILEY-V C H VERLAG GMBH. (**b**) Response of the CMC-fiber to humidity. Reproduced with permission [109]. Copyright 2018. ELSEVIER. (**c**) Photographs showing the shape recovery test of CNF/CMCNF9 bilayer film in DMSO and EtOH. Reproduced with permission [38]. Copyright 2030. SPRINGER. (**d**) Curling displacement of the CNF-soft actuator during the dehydration process; insets are the corresponding snapshots at different states. The numbers 1–5 indicated the different bending states of the samples. Reproduced with permission [110]. Copyright 2019. ELSEVIER. (**e**) Schematic of the RCF/PTFE composite film. (**f**) Ptical photos of the RCF/PTFE actuator at a low RH of 29% (**left panel**), room RH of 50% (**middle panel**), and high RH of 54% (**right panel**). Reproduced with permission [111]. Copyright 2021. ELSEVIER.

Ge et al. [108] reported a photonic film based on cellulose nanocrystals (CNCS), in which poly (ethylene glycol) dimethacrylate (PEGDMA) converges into the chiral nematic structure of CNC through UV-triggered free radical polymerization in a N, N-dimethylformamide solvent system. When the vertically fixed-size photonic film is exposed to moisture on its left side for 4 s, the vertical bending angle of the composite film increases to 135 °, and the bending rate is 4.6 °s^−1^, as shown in Figure 12a. Then, the moisture was removed and the film returned to its original vertical position for about 10 s. They further studied the reversibility of the film’s actuation and found that it exhibited excellent stability and reversibility after alternating exposure to moisture on both sides of the film for 20 cycles. Matthew et al. [109] used the humidity-driven volume change in carboxymethyl cellulose (CMC) to manufacture the humidity response actuator on the glass fiber substrate. As shown in Figure 12b, CMC-coated fiber showed a linear dependence on humidity in the range of 5–40% relative humidity, and the response time was 1 min. Cellulose nanofibers (CNFs) are obtained through the mechanical fibrillation of fibers. Due to their high aspect ratio, they facilitate the formation of intercalation layers of water molecules in the fiber network. Therefore, using CNFs as a substrate can significantly enhance their actuation performance. As shown in Figure 12c, Lisa et al. [38] prepared thin films based on cellulose nanofibers (CNFs), which undergo varying degrees of distortion and expansion when in contact with water or organic solvents. Kuang et al. [110] designed a substrate micropattern actuator based on selectively arranged cellulose nanofibers (CNFs). Due to the presence of more intermolecular hydrogen bonds (H bonds) between CNFs, reversible fracture and recombination can occur under the erosion of water molecules, resulting in good controllability, fast response time (less than 1 s), and curl displacement actuation during dehydration, as shown in Figure 12d.

Now, with the in-depth research of transparent materials, and due to their unique transparency and invisibility, they have been widely used in stealth robots, biomimetic skin, and other fields. Transparent actuators have also been extensively studied. Li et al. [111] reported a transparent cellulose-based actuator driven by humidity and infrared (IR) light, which is made of two layers of regenerated cellulose film (RCF) and hydrophobic polytetrafluoroethylene (PTFE) film, as shown in Figure 12e. The principle of its actuation is that RCF is hydrophilic (due to the presence of abundant hydroxyl groups), while PTFE film is hydrophobic. This difference leads to the asymmetry of the bilayer membrane structure, thereby obtaining internal stress that responds to the changes in environmental humidity. When the relative humidity decreases to 29%, the RCF/PTFE actuator bends towards the side of the RCF film, with a maximum bending curvature of 2.5 cm. Then, the rate of curvature increase slows down. When the relative humidity increases to 50%, the actuator returns to its original flat state. On the contrary, when the relative humidity increased to 54%, the RCF/PTFE actuator showed significant bending towards the PTFE film side, as shown in Figure 12f.

**Figure 13:**
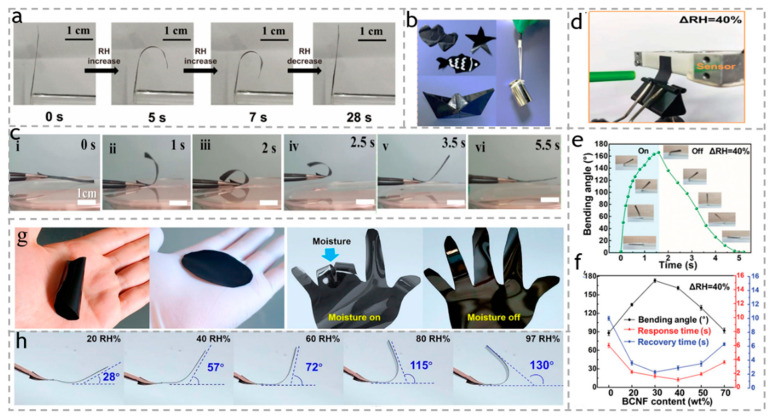
(**a**) Optical images of reversible hygroscopic bending actuation of the G-MXCP actuator under a relative humidity (RH) change from 40% to 90% at room temperature. Reproduced with permission [112]. Copyright 2020. AMER CHEMICAL SOC. (**b**) Digital pictures of CMC/MXene/Al^3+^ composite film, showing the excellent flexibility and mechanical strength. (**c**) The reversible deformation of CMC/MXene/Al^3+^ composite film upon exposure to humidity gradients. The sequential numbers i–vi indicated the sample with the different bending states. Reproduced with permission [113]. Copyright 2022. ELSEVIER. (**d**) Photographs of hygroscopic actuation force test of PDMM/BCNF film. (**e**) Bending angle–time curve of the PDMM/BCNF film (30 wt% BCNF) during a cyclic motion. (**f**) Bending angle and response/recovery time of PDMM/BCNF film with different BCNF content. Reproduced with permission [114]. Copyright 2021. ELSEVIER. (**g**) Optical images of an MCPM on a human hand with or without wearing a dry glove. (**h**) The maximum bending angles of an MCPM/PET actuator under various RH. Reproduced with permission [115]. Copyright 2021. American Chemical Society.

Combining high-performance materials with traditional environmentally friendly cellulose-based materials is a universal strategy for developing intelligent, green materials and flexible equipment. Transition metal carbides including carbonitrides and nitrides (MXene) are two-dimensional layered materials with excellent photothermal conversion capabilities and rich surface-chemical properties. As shown in Figure 13a, Cao et al. [112] reported a two-dimensional MXenes/cellulose nanofiber (CNF)/biomacromolecule soft actuator using a bio-inspired mesoscale assembly strategy that combines nano-, micro-, and macro-length scales. When humidity increased, the actuator exhibited a direct rapid actuation rate of 34.2 °s^−1^. Under infrared light (IR) heating, the actuator’s hygroscopicity and actuation performance were significantly improved. Inspired by the structure of the pearl layer of brick mortar, Wei et al. [113] prepared high-performance carboxymethyl cellulose-based actuators. MXene nanosheets and Al^3+^ were introduced into the CMC matrix through H and ion bonds, forming a multi-level pearl layer structure. The good wet strength in a high humidity environment is 2.97 MPa, with impressive flexibility, as shown in Figure 13b. More interestingly, due to the sensitive hygroscopicity of CMC molecules and MXene nanosheets, as shown in Figure 13c, the composite film can also exhibit a large bending angle (>180°), rapid response/recovery (2.3/3.0 s), and excellent cycling stability over 1500 cycles in response to humidity gradients, and simultaneously with reversible deformation. Yang et al. [114] prepared pearl-layered and layered composite films composed of MXene modified with polydopamine (PDA), and bacterial cellulose nanofibers, as shown in Figure 13d. The actuator has high conductivity (2848 S cm^−1^), excellent tensile strength (406 MPa), and toughness (15.3 MJ m^−3^). In addition, the actuator is highly sensitive to moisture (30% humidity), has a `fast response (1.6 s), large deformation (176 °), and high driving power output (6.5 N m^−2^), as shown in Figure 13e,f. Li et al. [115] prepared MXene/cellulose/polystyrene sulfonic acid (PSSA) composite membranes (MCPM), as shown in Figure 13g, which are highly sensitive to moisture and roll up immediately upon contact with the skin. When the relative humidity increases from 20% to 97%, the maximum bending angle of the thick MCPM actuator has increased from 28° to 130°, providing high sensitivity for humidity sensing, as shown in Figure 13h.

## 9. Application of Cellulose-Based Actuators

From the previous chapter, it can be observed that the driving forces of the cellulose-based actuators are diverse, and can even achieve multi stimulus responses. The combination of these characteristics makes the actuator have a wide range of application potential in the field of intelligence. In this chapter, the applications of four types of cellulose actuators are presented including biomimetic robots, flexible electronic devices, self-powered intelligent switches, and precise remote control.

### 9.1. Biomimetic Robots

Currently, the usage scenarios of robots are gradually tending towards precision, narrowness, and complexity. The demand for this task scenario will inevitably accelerate the transformation of biomimetic robots towards miniaturization. Through highly integrated components such as actuators and sensors, their overall miniaturization can be achieved. At the same time, the profiling of biomimetic robots has also become the main development trend.

**Figure 14:**
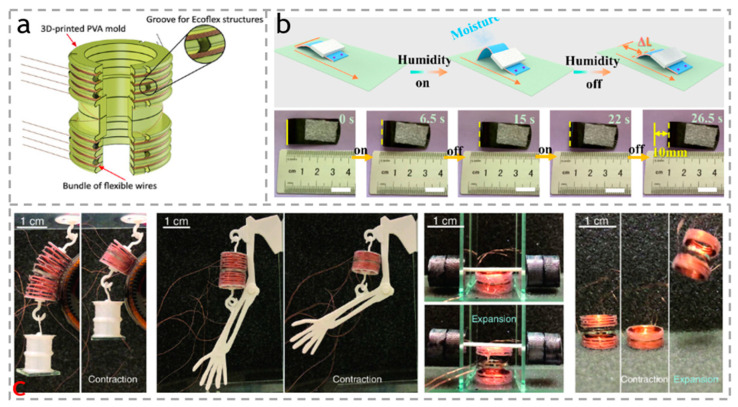
(**a**) Schematic of the soft actuator fabrication. Reproduced with permission [116]. Copyright 2023. WILEY. (**b**) Load-bearing smart robot crawling forward on a rough substrate under periodic humidity on and off. Reproduced with permission [117]. Copyright 2021. American Chemical Society. (**c**) Relaxed and excited states of a 12-ring actuator when lifting a 2 g weight while conforming to a cylindrically shaped object. Relaxed and excited states of a 12-ring actuator when used as an artificial muscle. Relaxed and excited states of an 8-ring actuator when lifting a 1.6 g weight by expansion. Hopping mode of actuation for an 8-ring actuator by contraction and subsequent expansion. Reproduced with permission [116]. Copyright 2023. WILEY.

Deforming the actuator and lifting heavy objects is currently the most widely studied and applied method, demonstrating excellent mechanical robustness. As shown in Figure 14a, Mu et al. [116] prepared multifunctional soft electromagnetic actuators (copper wire coils and central circular magnets) that can contract, expand, jump, and move. As shown in Figure 14b, Wei et al. [117] prepared CNF/GO/CNT composite membrane actuators (2 cm × 3 cm × 12 μm) containing 50% carbon nanotubes. Used as a crawling robot, it can gradually crawl forward on rough substrates in response to periodic water vapor changes. Interestingly, crawling robots can move heavy objects forward at an average speed of 2.6 mm/s through alternating humidity switches, which is likely to be used for crawling robots or humidity control switches. As shown in Figure 14c, the actuator has a good contact with the cylinder and repeatedly lifts a weight of 2 g under different currents and strains, perfectly demonstrating its operating ability under irregular geometric shapes. It can not only be used as an artificial muscle to make 3D printed polylactic acid (PLA) arms move normally, but can also lift a weight of 2 g weight in its expanded state. According to its two states (expansion and contraction), it can jump in the air at a height several times its own height. Finally, they use it as an attachment to the leg, where the actuator can crawl along the structured surface, crawling like a human leg. Kuang et al. [110] designed a micro-patterned substrate actuator based on selectively arranged cellulose nanofibers (CNFs). They made it into a 7 cm soft mechanical arm. After heating, the actuator strip quickly curled. With the length contraction, the attached object was lifted up. When the humidity in the environment was increased, the actuator strip quickly unfolded, leading to the release of suspended weights.

**Figure 15:**
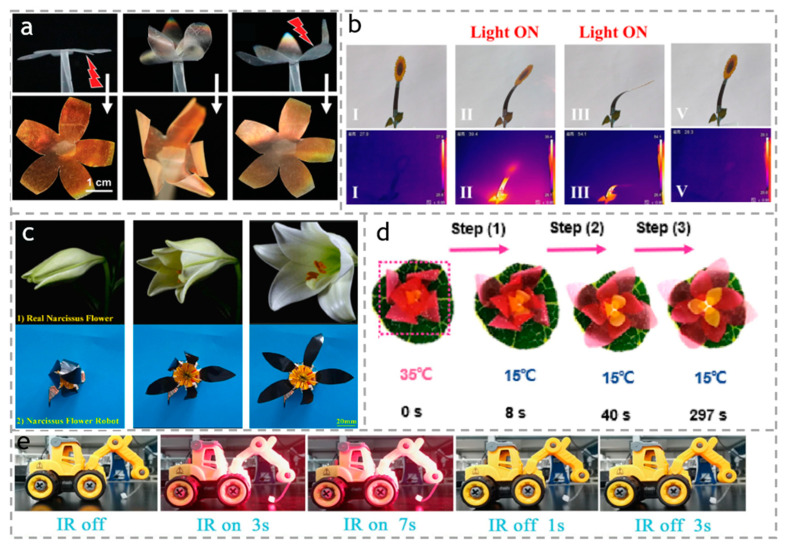
(**a**) Closing and opening on an artificial “flower” by moisture stimulation. Reproduced with permission [108]. Copyright 2022. WILEY-VCHVERLAG GMBH. (**b**) Optical photos of infrared thermal images. The sub-images I–III, V indicated the different states of the samples under the light on and off. Reproduced with permission [118]. Copyright 2022. Royal Society of Chemistry. (**c**) Petal opening of a bionic flower. Petal opening of a bionic flower. Reproduced with permission [119]. Copyright 2023. ELSEVIER. (**d**) The blooming of the lotus flower of ABH actuator. The red dots indicated the flower sample. The arrows indicated the blooming process. Reproduced with permission [44]. Copyright 2021, AMER CHEMICAL SOC. (**e**) Series of optical photographs demonstrating the arm of an excavator-shape actuator responding to IR light. Reproduced with permission [122]. Copyright 2023. ELSEVIER.

Many researchers have been inspired by nature to create petal-shaped actuators that simulate the opening and closing of petals. Ge et al. [108] prepared flower-shaped actuators based on cellulose nanocrystals. As shown in Figure 15a, under the stimulation of water, the petals not only produce bending responses but also undergo corresponding color changes. As shown in Figure 15b, Yang et al. [118] used a GO-BC/PET/PEDOT: PSS actuator as the stem of a sunflower. After 15 s of near-infrared light exposure, the temperature rapidly increased, causing the biomimetic sunflower to bend towards the light. More interestingly, Wang et al. [119] simulated the opening and closing process of narcissus petals. As shown in Figure 15c, they simulated the opening and closing process of the petals under a sinusoidal voltage of 0 V and 1.8 Hz, and changed from a closed state to open state in 2 s, based on the fast electric response ionic actuator of the functional cellulose carboxylate nanofiber (CCNF) doped ionic liquid (IL), and Graphene nanosheet (GN). When the input voltage stops, the bionic flower can recover its state of nature in a short time. Cai et al. [120] innovatively crafted a composite material, consisting of MXene (Ti_3_C_2_Tx) and cellulose, referred to as MXCC. Alongside a polycarbonate membrane, the composite replicates multiple essential characteristics of natural leaves, ranging from microstructural components to their photosynthetic abilities, encompassing energy harvesting and conversion. In addition, Chen et al. [44] prepared bamboo/PNIPAM composite hydrogel actuators. As shown in Figure 15d, they used cutouts of different thicknesses and sizes of actuators as the petals of a lotus flower. As the temperature decreases, the lotus flower continuously blooms from the outside to the inside.

The biomimetic actuators discussed above have an inherent drawback of being opaque, limiting their use in discreet tasks. This has led to a growing interest among researchers to develop transparent actuators that offer high concealment. Such advancements in technology would enable the design of biomimetic animals, windows, and robots that can execute covert operations without being easily detected by humans. Rühlicke et al. [121] devised a cutting-edge cellulose blend with distinctive capabilities in specific environments, leading to the development of a clear, thin film actuator with the ability to self-heal and undergo shape deformation. On another note, as shown in Figure 15e, Li et al. [122] successfully created a cellulose composite thin film that is both visible and transparent, while also displaying a remarkable capacity for absorbing ultraviolet light. The actuator designed by Li et al. was modeled after the arm of an excavator and proved to be highly efficient, as it was able to lift an object 17 times its weight swiftly when exposed to infrared light. Upon turning off the light source, the object fell rapidly.

### 9.2. Flexible Electronic Equipment

Flexible electronic devices with high mechanical performance have been widely used in a large number of wearable/implantable devices, greatly enriching the collection of health data and disease detections. However, due to the monopoly of its core technology and the expensive raw materials, a main direction to search for simple, cost-effective and biodegradable technique is highly desirable.

**Figure 16:**
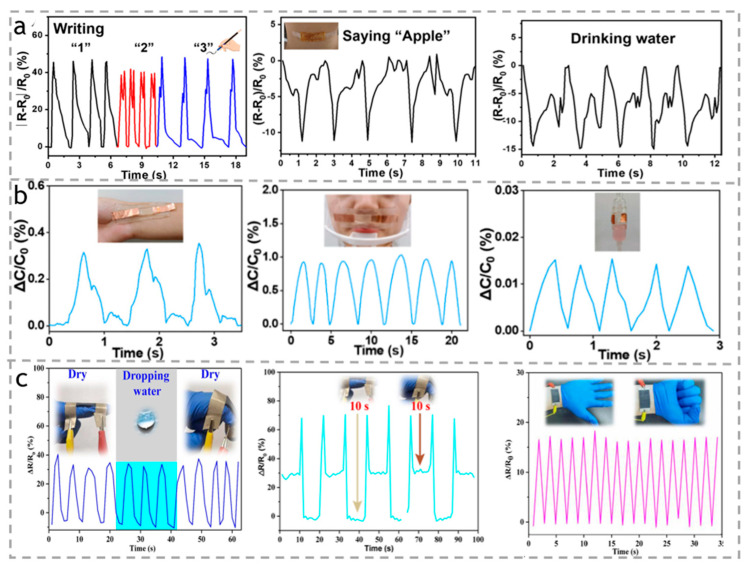
(**a**) The relative resistance changes by writing a different number, saying “apple”, and drinking water. Reproduced with permission [123]. Copyright 2023. ELSEVIER. (**b**) The corresponding pulse signals, breathing in relaxation, and the drip pot’s little vibration when liquid drips into it. Reproduced with permission [124]. Copyright 2023. ROYAL SOC CHEMISTRY. (**c**) The resistance output changes in the state of finger movement. Insets: the PB strain sensor attached to the finger was repeatedly bent under normal conditions and in the presence of water droplets. The resistance output changes of periodically bending the index finger at a fixed angle, with fist opening and closing. Reproduced with permission [125]. Copyright 2023. ELSEVIER.

Skin conductivity is a key feature in the design of electronic watches. By monitoring the pulse and other physiological indicators of the skin, these smart watches can provide accurate health data, helping users better to understand their physical conditions. As shown in Figure 16a, Lian et al. [123] have developed a cellulose-based ion-conductive hydrogel actuator (ICHs) using a simple one-pot method. This actuator is capable of accurately detecting the different pressure signals when humans write different numbers. Surprisingly, apart from monitoring complex finger movements, the actuator can also monitor complex muscle movements. In an experiment, participants were asked to say “apple” while drinking water, and the actuator detected different resistance signals by sensing the weak acoustic vibrations and water vibrations. This demonstrates the reliability of cellulose hydrogel actuators in detecting both sound signals and actions. Similarly, in order to improve the high sensitivity of the flexible pressure sensor, as shown in Figure 16b, Chen et al. [124] designed a cellulose ion-conductive hydrogel (ICH) using only cellulose as the sole material. They simulated the scenario of patient infusion and placed the sensor at different positions to monitor the patient’s health status by detecting physiological signals such as pulse, respiration, and infusion.

The above actuators did not consider the water resistance of the sensors. Yun et al. [125] developed a superhydrophobic and highly sensitive cellulose paper-based actuator (PB) that can quickly detect and perceive the bending of a finger. Impressively, the continuous dripping of water does not affect the sensing performance, which is advantageous for applications in wet or rainy conditions, as shown in Figure 16c.

Electrospinning is a simple and efficient fiber preparation method [126,127], and has various applications in different fields [128,129,130]. Electrospinning technology can integrate actuation and sensing functions into a functional material. It is understood that electrospun fibrous membrane has the characteristics of a large surface area, multi-level porous structure, strong flexibility, and easy shape control [131,132,133,134]. Therefore, Bai et al. [73] used electrospun cellulose acetate (CA)/carbon nanotube nanofiber composite membrane to prepare a friction generator with a polyvinylidene fluoride nanofiber membrane (PVDF). This membrane can detect the human movement of fingers, wrists, elbows, and other joints. In addition, when it is installed on the sole of the shoe, people can distinguish the walking state (slow walking and running) by the change of voltage and frequency.

### 9.3. Accurate Remote Control

With the rapid development of modern society’s technology, mechanical equipment plays an important role in the development of society’s technology. However, most construction machinery requires manual operation by skilled workers. Many workplaces are mostly high-risk or toxic environments, and the remote control of mechanical equipment is particularly important.

**Figure 17:**
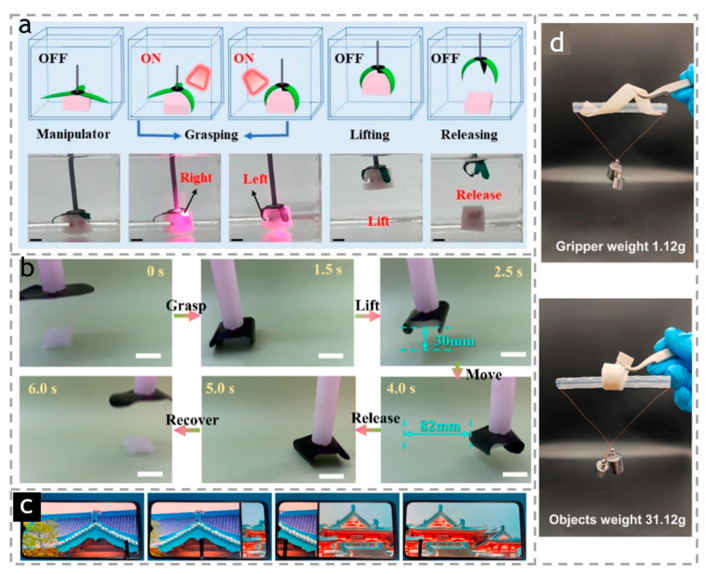
(**a**) Demonstration of precise control performance of bamboo/PNIPAM composite hydrogel actuator-manipulator grasping object. Reproduced with permission [84,85]. Copyright 2022. ELSEVIER. (**b**) Schematic illustration and photographs of the mechanical gripper for transferring a plastic foam. Reproduced with permission [84,85]. Copyright 2022. ELSEVIER. (**c**) A bionic finger sliding photos on the electronic screen. Reproduced with permission [135]. Copyright 2023. SPRINGER. (**d**) Soft grippers withstanding 30 times their weight. Reproduced with permission [136]. Copyright 2023. ELSEVIER.

The bamboo/PNIPAM composite hydrogel actuator prepared by Chen et al. [84,85] is designed as a manipulator with three claws for fixed-point control, as shown in Figure 17a. It can lift objects that are dozens of times heavier than the actuator under the control of near-infrared light. They place the manipulator in water, irradiate the right paw, left paw, and rear paw with infrared light in sequence. The paws shrink in sequence, and start to grasp the heavy objects. After turning off the light source, the paws return to its original shape; as a result, the heavy object falls down, which perfectly demonstrates the precise remote control. Similarly, they also made a wood/PNIPAM composite hydrogel actuator [84,85]. Through a similar operation, it was also proved that the actuator can easily grasp and place objects according to external changes. Wei et al. [117] prepared CNF/GO/CNT composite membrane actuators containing 50% carbon nanotubes. When the actuator was connected to a plastic straw, the resultant simple gripper can perform tasks such as grasping, lifting, transporting, and releasing according to the changes in humidity, as shown in Figure 17b. The plastic foam can be even be bent and grasped by applying water vapor through the plastic straw. Then, the holder can lift the foam (about 0.12 g, 9 times of the actuator) and carry 82 mm horizontally under the stimulation of continuous water vapor. Finally, when the water vapor is removed, the gripper can quickly release foam. Such an actuator is expected to be applied to mechanical grippers due to its large deformation, fast response time, and good cycle stability.

In addition to accurately grasping heavy objects, people have even studied using actuators as human fingers to control smartphones. Wang et al. [135] used CCNF-IL-GN actuators to simulate human fingers sliding on smartphones and switching photos. When a 4 V DC voltage is applied, the bionic fingers move on the track to touch the screen, as shown in Figure 17c, causing them to slide through albums. In addition, the bionic fingers can also accurately control the opening or closing status of flashlights. This high-performance bionic actuator can be used in the field of artificial muscle.

Shape memory polymers (SMPs) have found extensive application in the field of 3D printing. Furthermore, with advancements in intelligent deformations, 4D printing has begun to mature. Gu et al. [136] performed an experiment to showcase soft grippers that were inspired by plants and achieved through 4D printing with different filling angles. They utilized a straightforward fabrication approach to create thermo-responsive shape memory polymer composites by reinforcing poly ε-caprolactone (PCL)/polybutylene adipate-co-terephthalate (PBAT) with cellulose nanofibers (CNF). By subjecting the samples to thermal stimulation at a temperature of 3 °C, they observed a rapid deformation and wrapping around objects within 60 s. These spiral and bending deformations enabled the grippers to hold objects weighing up to 30 times their own weight, as shown in Figure 17d.

## 10. Conclusions and Future Prospects

With the aggravation of the energy crisis and environmental degradation, the development and utilization of renewable biomass resources has become the main content of research worldwide [137,138,139,140,141]. The development and utilization of cellulose, the most abundant biomass resource in nature, in order to build new materials, conforms to the future development trend. This article reviews the latest progress and achievements of cellulose-based actuators, discussing them from three aspects: actuation mechanism, driving force, and applications. Due to the significant advantages of cellulose itself, temperature-sensitive materials, conductive materials, and magnetic materials are often introduced to endow cellulose-based materials with different functions, to promote the speed and stability of their response to external stimuli. This has wide applications in fields such as biomimetic robots, flexible electronic wearable devices, and remote control.

There has been significant progress in cellulose-based actuators, but there are still some challenges. Firstly, cellulose fiber, as a textile fiber, has long permeated people’s daily lives. However, fibers produced by the adhesive method still dominate the market, requiring the use of various toxic and harmful substances in the production process, causing great environmental pollution. The further development and improvement of production processes of cellulose fibers is highly required.

Secondly, the lifespan of actuators that are produced through cellulose degradation is relatively short. Moreover, biomimetic materials are frequently derived from materials that are sensitive to environmental variations. While this characteristic may prove useful, it also limits their applicability in certain settings. Hydrogels are a notable example, as they necessitate an aqueous environment and cannot be utilized in dry applications. Presently produced actuators do not possess the ability to adapt to intricate environments. Consequently, it is recommended to conduct further research into their multifunctional capabilities, such as luminescence, anti-freezing properties, signal sensing, and adsorption.

Finally, although functional materials based on cellulose have shown promising practical applications, most of them are still in the stage of basic research and are limited in size, which hinders their practical applications on a large scale. Further research and development is required to enhance the experimental equipment, broaden the scope of applications, and promote practical utilization, thereby creating substantial economic and social benefits.

Despite the aforementioned limitations, cellulose-based actuators present several advantages such as low production cost, utilization of eco-friendly raw materials, and versatility in various fields such as bionic robots, wearable electronic devices, and remote-controlled gripping. Consequently, with the swift advancement of technology, the research on cellulose-based actuators is steadily progressing towards maturity, and their incorporation into flexible electronic devices will undoubtedly take center stage in the forthcoming years.

## Data Availability

The data presented in this study are available on request from the corresponding author. The data are not publicly available due to the demand from our further research.

## References

[1] Wu Y., Li Y., Tao Y., Sun L., Yu C. (2023). Recent advances in the material design for intelligent wearable devices. Mater. Chem. Front..

[2] Wang J., Li S., Gao D., Xiong J., Lee P.S. (2019). Reconfigurable and programmable origami dielectric elastomer actuators with 3D shape morphing and emissive architectures. NPG Asia Mater..

[3] Wang F., Wang L., Wang Y., Wang D. (2022). Highly bendable ionic electroactive polymer actuator based on carboxylated bacterial cellulose by doping with MWCNT. Appl. Phys. A.

[4] Nevstrueva D., Murashko K., Vunder V., Aabloo A., Pihlajamäki A., Mänttäri M., Pyrhönen J., Koiranen T., Torop J. (2018). Natural cellulose ionogels for soft artificial muscles. Colloids Surf. B Biointerfaces.

[5] Chen W., Sun B., Biehl P., Zhang K. (2022). Cellulose-Based Soft Actuators. Macromol. Mater. Eng..

[6] Constantinos M., Pfeiffer C., Mosley M. (1999). Conventional actuators, shape memory alloys and electrorheological fluids. Autom. Miniat. Robot. Sens. Non-Destr. Test. Eval..

[7] Al-Khalili J. (2015). The birth of the electric machines: A commentary on Faraday (1832) ‘Experimental researches in electricity’. Philos. Trans. R. Soc. A Math. Phys. Eng. Sci..

[8] Yu Y., Wang J., Han X., Yang S., An G., Lu C. (2023). Fiber-Shaped Soft Actuators: Fabrication, Actuation Mechanism and Application. Adv. Fiber Mater..

[9] Kim H., Lee J.A., Ambulo C.P., Lee H.B., Kim S.H., Naik V.V., Haines C.S., Aliev A.E., Ovalle-Robles R., Baughman R.H. (2019). Intelligently Actuating Liquid Crystal Elastomer-Carbon Nanotube Composites. Adv. Funct. Mater..

[10] Hwang I., Kim H.J., Mun S., Yun S., Kang T.J. (2021). A Light-Driven Vibrotactile Actuator with a Polymer Bimorph Film for Localized Haptic Rendering. ACS Appl. Mater. Interfaces.

[11] Xiong R., Sauvage F., Fraire J.C., Huang C., De Smedt S.C., Braeckmans K. (2023). Photothermal Nanomaterial-Mediated Photoporation. Acc. Chem. Res..

[12] Chen L., Weng M., Huang F., Zhang W. (2019). Light- and humidity-driven actuators with programmable complex shape-deformations. Sens. Actuators B Chem..

[13] Bhatti M.R.A., Bilotti E., Zhang H., Varghese S., Verpaalen R.C.P., Schenning A., Bastiaansen C.W.M., Peijs T. (2020). Ultra-High Actuation Stress Polymer Actuators as Light-Driven Artificial Muscles. ACS Appl. Mater. Interfaces.

[14] Han D.D., Zhang Y.L., Ma J.N., Liu Y.Q., Han B., Sun H.B. (2016). Light-Mediated Manufacture and Manipulation of Actuators. Adv. Mater..

[15] Quashie D., Benhal P., Chen Z., Wang Z., Mu X., Song X., Jiang T., Zhong Y., Cheang U.K., Ali J. (2022). Magnetic bio-hybrid micro actuators. Nanoscale.

[16] Xu H., Xu X., Xu J., Dai S., Dong X., Han F., Yuan N., Ding J. (2019). An ultra-large deformation bidirectional actuator based on a carbon nanotube/PDMS composite and a chitosan film. J. Mater. Chem. B.

[17] Miriyev A., Stack K., Lipson H. (2017). Soft material for soft actuators. Nat Commun.

[18] Yang H., Leow W.R., Wang T., Wang J., Yu J., He K., Qi D., Wan C., Chen X. (2017). 3D Printed Photoresponsive Devices Based on Shape Memory Composites. Adv. Mater..

[19] Zhao P., Xu B., Zhang Y., Li B., Chen H. (2020). Study on the Twisted and Coiled Polymer Actuator with Strain Self-Sensing Ability. ACS Appl. Mater. Interfaces.

[20] Liu W., Cheng Y., Liu N., Yue Y., Lei D., Su T., Zhu M., Zhang Z., Zeng W., Guo H. (2021). Bionic MXene actuator with multiresponsive modes. Chem. Eng. J..

[21] Melling D., Martinez J.G., Jager E.W.H. (2019). Conjugated Polymer Actuators and Devices: Progress and Opportunities. Adv. Mater..

[22] Wehner M., Truby R.L., Fitzgerald D.J., Mosadegh B., Whitesides G.M., Lewis J.A., Wood R.J. (2016). An integrated design and fabrication strategy for entirely soft, autonomous robots. Nature.

[23] Basu S., Omadjela O., Gaddes D., Tadigadapa S., Zimmer J., Catchmark J.M. (2016). Cellulose Microfibril Formation by Surface-Tethered Cellulose Synthase Enzymes. ACS Nano.

[24] Sinha A.K., Bhattacharya S., Narang H.K. (2020). Abaca fibre reinforced polymer composites: A review. J. Mater. Sci..

[25] Turner S., Kumar M. (2018). Cellulose synthase complex organization and cellulose microfibril structure. Philos. Trans. R. Soc. A Math. Phys. Eng. Sci..

[26] Che X., Wu M., Yu G., Liu C., Xu H., Li B., Li C. (2022). Bio-inspired water resistant and fast multi-responsive Janus actuator assembled by cellulose nanopaper and graphene with lignin adhesion. Chem. Eng. J..

[27] Zhang D., Jian J., Xie Y., Gao S., Ling Z., Lai C., Wang J., Wang C., Chu F., Dumont M.-J. (2022). Mimicking skin cellulose hydrogels for sensor applications. Chem. Eng. J..

[28] Yang B., Bi W., Zhong C.a., Huang M., Ni Y., He L., Wu C. (2018). Moisture-triggered actuator and detector with high-performance: Interface engineering of graphene oxide/ethyl cellulose. Sci. China Mater..

[29] Lin F., Wang Z., Shen Y., Tang L., Zhang P., Wang Y., Chen Y., Huang B., Lu B. (2019). Natural skin-inspired versatile cellulose biomimetic hydrogels. J. Mater. Chem. A.

[30] Khan A., Khan F.R., Kim H.S. (2018). Electro-Active Paper as a Flexible Mechanical Sensor, Actuator and Energy Harvesting Transducer: A Review. Sensors.

[31] Lee S.-W., Kim J.-H., Kim J., Kim H.S. (2009). Characterization and sensor application of cellulose electro-active paper (EAPap). Chin. Sci. Bull..

[32] Yang H., Wu F., Zhu G., Li H., Jiang S. (2023). Recent progress of modification and industrialization for nanocellulose towards green building materials. J. For. Eng..

[33] Liang X., Chen G., Lin S., Zhang J., Wang L., Zhang P., Lan Y., Liu J. (2022). Bioinspired 2D Isotropically Fatigue-Resistant Hydrogels. Adv. Mater..

[34] Sano K., Ishida Y., Aida T. (2018). Synthesis of Anisotropic Hydrogels and Their Applications. Angew. Chem. Int. Ed..

[35] Roopsung N., Sugawara A., Hsu Y.I., Asoh T.A., Uyama H. (2023). Cellulose Nanocrystal-Based Gradient Hydrogel Actuators with Controllable Bending Properties. Macromol. Rapid Commun..

[36] Schafer J.L., Meckel T., Poppinga S., Biesalski M. (2023). Chemical Gradients in Polymer-Modified Paper Sheets-Towards Single-Layer Biomimetic Soft Robots. Biomimetics.

[37] Wang X., Han B., Yu R.-P., Li F.-C., Zhao Z.-Y., Zhang Q.-C., Lu T.J. (2018). Magnetic-responsive Fe3O4 nanoparticle-impregnated cellulose paper actuators. Extrem. Mech. Lett..

[38] Lopes da Costa L., Moreau C., Lourdin D., Cathala B., Villares A. (2023). Shape-recovery in organic solvents of water-responsive cellulose nanofiber actuators. Cellulose.

[39] Maki Y. (2019). Poly(N,N-dimethylacrylamide)-clay nanocomposite hydrogels with patterned mechanical properties. Colloid Polym. Sci..

[40] Zhang E., Wang T., Hong W., Sun W., Liu X., Tong Z. (2014). Infrared-driving actuation based on bilayer graphene oxide-poly(N-isopropylacrylamide) nanocomposite hydrogels. J. Mater. Chem. A.

[41] Liu L., Jiang S., Sun Y., Agarwal S. (2015). Giving Direction to Motion and Surface with Ultra-Fast Speed Using Oriented Hydrogel Fibers. Adv. Funct. Mater..

[42] Ma C., Li T., Zhao Q., Yang X., Wu J., Luo Y., Xie T. (2014). Supramolecular Lego Assembly Towards Three-Dimensional Multi-Responsive Hydrogels. Adv. Mater..

[43] Wu Q., Ma C., Chen L., Sun Y., Wei X., Ma C., Zhao H., Yang X., Ma X., Zhang C. (2022). A Tissue Paper/Hydrogel Composite Light-Responsive Biomimetic Actuator Fabricated by In Situ Polymerization. Polymers.

[44] Chen L., Zhang K., Ahn J., Wang F., Sun Y., Lee J., Young Cheong J., Ma C., Zhao H., Duan G. (2023). Morph-genetic bamboo-reinforced hydrogel complex for bio-mimetic actuator. Chem. Eng. J..

[45] Huang B., Lin F., Tang L., Lu Q., Lu B. (2022). Research advances of functional cellulose-based hydrogels and its applications. J. For. Eng..

[46] Wu Y., Minamikawa H., Nakazumi T., Hara Y. (2020). Actuation Properties of Paper Actuators Fabricated Using PEDOT/PSS Electrode Films. J. Oleo. Sci..

[47] Terasawa N. (2021). High-performance TEMPO-oxidised cellulose nanofibre/PEDOT:PSS/ionic liquid gel actuators. Sens. Actuators B Chem..

[48] Chen Y., Kuang P., Shen X., Lv X., Wang Y., Yin W., Zou T., Wang B., Liu Y., Fan Q. (2023). Lignin precipitation-driven fabrication of gradient porous hydrogel actuator with temperature response. Smart Mater. Struct..

[49] Gao S., Zhou A., Cao B., Wang J., Li F., Tang G., Jiang Z., Yang A., Xiong R., Lei J. (2021). A tunable temperature-responsive and tough platform for controlled drug delivery. New J. Chem..

[50] Ghosh R., Misra A. (2021). Carbon Nanotube-Based Hierarchical Paper Structure for Ultra-high Electrothermal Actuation in a Wide Humidity Range. ACS Appl. Electron. Mater..

[51] Wei Y., Huo H., Huang C., Zhang Q., Hoogenboom R., Liu F. (2020). Supramolecular control over self-assembly and double thermoresponsive behavior of an amphiphilic block copolymer. Eur. Polym. J..

[52] Toron B., Szperlich P., Koziol M. (2020). SbSI Composites Based on Epoxy Resin and Cellulose for Energy Harvesting and Sensors-The Influence of SBSI Nanowires Conglomeration on Piezoelectric Properties. Materials.

[53] Liu S., Yu B., Wang S., Shen Y., Cong H. (2020). Preparation, surface functionalization and application of Fe(3)O(4) magnetic nanoparticles. Adv. Colloid Interface Sci..

[54] Vivekananthan V., Chandrasekhar A., Alluri N.R., Purusothaman Y., Khandelwal G., Pandey R., Kim S.-J. (2019). Fe2O3 magnetic particles derived triboelectric-electromagnetic hybrid generator for zero-power consuming seismic detection. Nano Energy.

[55] Xu W., Zheng X., Shangguan Z., Qu J., Zhang W. (2023). A low-cost magnetic catalyst (MnFe2O4) for ciprofloxacin degradation via periodate activation: The synergistic effect of Mn and Fe. Chem. Eng. J..

[56] Erdem D., Bingham N.S., Heiligtag F.J., Pilet N., Warnicke P., Heyderman L.J., Niederberger M. (2016). CoFe2O4and CoFe2O4-SiO2Nanoparticle Thin Films with Perpendicular Magnetic Anisotropy for Magnetic and Magneto-Optical Applications. Adv. Funct. Mater..

[57] Li Z., Wang J., Xu Y., Shen M., Duan C., Dai L., Ni Y. (2021). Green and sustainable cellulose-derived humidity sensors: A review. Carbohydr. Polym..

[58] Zhou S., Kong X., Strømme M., Xu C. (2022). Efficient Solar Thermal Energy Conversion and Utilization by a Film of Conductive Metal–Organic Framework Layered on Nanocellulose. ACS Mater. Lett..

[59] Tian Y., Liu Y., Ju B., Ren X., Dai M. (2019). Thermoresponsive 2-hydroxy-3-isopropoxypropyl hydroxyethyl cellulose with tunable LCST for drug delivery. RSC Adv..

[60] Mo K., He M., Cao X., Chang C. (2020). Direct current electric field induced gradient hydrogel actuators with rapid thermo-responsive performance as soft manipulators. J. Mater. Chem. C.

[61] Michalska-Walkowiak J., Forster B., Hauschild S., Forster S. (2022). Bistability, Remanence, Read/Write-Memory, and Logic Gate Function via a Stimuli-Responsive Polymer. Adv. Mater..

[62] Jiang S., Helfricht N., Papastavrou G., Greiner A., Agarwal S. (2018). Low-Density Self-Assembled Poly(N-Isopropyl Acrylamide) Sponges with Ultrahigh and Extremely Fast Water Uptake and Release. Macromol. Rapid Commun..

[63] Kajornprai T., Katesripongsa P., Nam S.Y., Hamid Z.A.A., Ruksakulpiwat Y., Suppakarn N., Trongsatitkul T. (2023). Potential Applications of Thermoresponsive Poly(N-Isoproplacrylamide)-Grafted Nylon Membranes: Effect of Grafting Yield and Architecture on Gating Performance. Polymers.

[64] Liu W., Geng L., Wu J., Huang A., Peng X. (2022). Highly strong and sensitive bilayer hydrogel actuators enhanced by cross-oriented nanocellulose networks. Compos. Sci. Technol..

[65] Wei Z., Liu Z., Wang X., Long S., Yang J. (2019). Smart carrier from electrospun core-shell thermo-sensitive ultrafine fibers for controlled drug release. Eur. Polym. J..

[66] Wang Y., Song S., Chu X., Feng W., Li J., Huang X., Zhou N., Shen J. (2021). A new temperature-responsive controlled-release pesticide formulation—*Poly*(N-isopropylacrylamide) modified graphene oxide as the nanocarrier for lambda-cyhalothrin delivery and their application in pesticide transportation. Colloids Surf. A Physicochem. Eng. Asp..

[67] Zhen C., Jing L., Yujie C., Xu Z., Hezhou L., Hua L. (2020). Multiple-Stimuli-Responsive and Cellulose Conductive Ionic Hydrogel for Smart Wearable Devices and Thermal Actuators. ACS Appl. Mater. Interfaces.

[68] Zong R., Hu X., Shang M., Wu C., Shentu B. (2023). Phase Morphology and Conductive Properties of PBT/POE-g-GMA/PP/CNT Nanocomposites with a Tri-Continuous Structure via Thermal Annealing. Ind. Eng. Chem. Res..

[69] Mao F., Fan X., Long L., Li Y., Chen H., Zhou W. (2023). Constructing 3D hierarchical CNTs/VO2 composite microspheres with superior electromagnetic absorption performance. Ceram. Int..

[70] Ji C., Wang Y., Ye Z., Tan L., Mao D., Zhao W., Zeng X., Yan C., Sun R., Kang D.J. (2020). Ice-Templated MXene/Ag-Epoxy Nanocomposites as High-Performance Thermal Management Materials. ACS Appl. Mater. Interfaces.

[71] Mao F., Long L., Pi W., Li Y., Zhou W. (2022). X-band electromagnetic absorption and mechanical properties of mullite/Ti3AlC2 composites. Mater. Chem. Phys..

[72] Wei J., Jia S., Wei J., Ma C., Shao Z. (2021). Tough and Multifunctional Composite Film Actuators Based on Cellulose Nanofibers toward Smart Wearables. ACS Appl. Mater. Interfaces.

[73] Bai Y., Zhou Z., Zhu Q., Lu S., Li Y., Ionov L. (2023). Electrospun cellulose acetate nanofibrous composites for multi-responsive shape memory actuators and self-powered pressure sensors. Carbohydr. Polym..

[74] Almeida A.P.C., Canejo J.P., Almeida P.L., Godinho M.H. (2019). Cholesteric-type cellulosic structures: From plants to applications. Liq. Cryst..

[75] Chang T., Wang B., Yuan D., Wang Y., Smalyukh I., Zhou G., Zhang Z. (2022). Cellulose nanocrystal chiral photonic micro-flakes for multilevel anti-counterfeiting and identification. Chem. Eng. J..

[76] Juanjuan S., Jiayin L., Weidong Y., Jialing T., Yunjie Y., Chaoxia W. (2022). Chiral Nematic Solvent-Responsive Actuator Based on a Cellulose Nanocrystal Template. ACS Appl. Polym. Mater..

[77] Li X., Liu J., Zhang X. (2023). Pressure/Temperature Dual-Responsive Cellulose Nanocrystal Hydrogels for On-Demand Schemochrome Patterning. Adv. Funct. Mater..

[78] Orelma H., Hokkanen A., Leppänen I., Kammiovirta K., Kapulainen M., Harlin A. (2019). Optical cellulose fiber made from regenerated cellulose and cellulose acetate for water sensor applications. Cellulose.

[79] Ren Y., Fang L., Liu Y., Shen C., Wu L., Zheng X., Fang K. (2022). Warming performance of far infrared lights electrogenerated by carbon nanotubes composite fabrics. Mater. Lett..

[80] Loeb S., Li C., Kim J.H. (2018). Solar Photothermal Disinfection using Broadband-Light Absorbing Gold Nanoparticles and Carbon Black. Env. Sci. Technol..

[81] Simayee M., Esfandiar A. (2022). Synergistic effect of reduced graphene oxide and carbon black as hybrid light absorber for efficient and antifouling texture-based solar steam generator. Sol. Energy.

[82] Zhao Q., Liang Y., Ren L., Yu Z., Zhang Z., Qiu F., Ren L. (2018). Design and fabrication of nanofibrillated cellulose-containing bilayer hydrogel actuators with temperature and near infrared laser responses. J. Mater. Chem. B.

[83] Xinkai L., Jize L., Dongdong L., Shaoquan H., Kai H., Xinxing Z. (2021). Bioinspired Multi-Stimuli Responsive Actuators with Synergistic Color- and Morphing-Change Abilities. Adv. Sci..

[84] Chen L., Wei X., Wang F., Jian S., Yang W., Ma C., Duan G., Jiang S. (2022). In-situ polymerization for mechanical strong composite actuators based on anisotropic wood and thermoresponsive polymer. Chin. Chem. Lett..

[85] Chen L., Wei X., Sun Y., Xue Y., Wang J., Wu Q., Ma C., Yang X., Duan G., Wang F. (2022). A bamboo/PNIPAM composite hydrogel assembly for both programmable and remotely-controlled light-responsive biomimetic actuations. Chem. Eng. J..

[86] Wang F., Lee J., Chen L., Zhang G., He S., Han J., Ahn J., Cheong J.Y., Jiang S., Kim I.-D. (2023). Inspired by Wood: Thick Electrodes for Supercapacitors. ACS Nano.

[87] Zhang Y., Xiao H., Xiong R., Huang C. (2023). Xylan-based ratiometric fluorescence carbon dots composite with delignified wood for highly efficient water purification and photothermal conversion. Sep. Purif. Technol..

[88] Qu Q., Zhang J., Chen X., Ravanbakhsh H., Tang G., Xiong R., Manshian B.B., Soenen S.J., Sauvage F., Braeckmans K. (2021). Triggered Release from Cellulose Microparticles Inspired by Wood Degradation by Fungi. ACS Sustain. Chem. Eng..

[89] Chen L., Sun Y., Wang J., Ma C., Peng S., Cao X., Yang L., Ma C., Duan G., Liu Z. (2022). A wood-mimetic porous MXene/gelatin hydrogel for electric field/sunlight bi-enhanced uranium adsorption. e-Polymers.

[90] Yun G.-Y., Kim J.-H., Kim J. (2009). Dielectric and polarization behaviour of cellulose electro-active paper (EAPap). J. Phys. D Appl. Phys..

[91] Irfan M., Ali I., Ali A., Ahmed M., Soomro T.A., Yang W., Rahman S., Faraj Mursal S.N., Jalalah M., Jazem Ghanim A.A. (2023). Analysis of the Performance of a Gel Actuator Made of Plasticized Polyvinyl Chloride/Carboxylated Cellulose Nanocrystals. ACS Omega.

[92] Zhu H., Dai W., Wang L., Yao C., Wang C., Gu B., Li D., He J. (2022). Electroactive Oxidized Alginate/Gelatin/MXene (Ti(3)C(2)T(x)) Composite Hydrogel with Improved Biocompatibility and Self-Healing Property. Polymers.

[93] Deng W., Xu Y., Zhang X., Li C., Liu Y., Xiang K., Chen H. (2022). (NH4)2Co2V10O28·16H2O/(NH4)2V10O25·8H2O heterostructure as cathode for high-performance aqueous Zn-ion batteries. J. Alloys Compd..

[94] Wu M.-S., Lyu L.-J., Syu J.-H. (2015). Copper and nickel hexacyanoferrate nanostructures with graphene-coated stainless steel sheets for electrochemical supercapacitors. J. Power Sources.

[95] Sen I., Seki Y., Sarikanat M., Cetin L., Gurses B.O., Ozdemir O., Yilmaz O.C., Sever K., Akar E., Mermer O. (2015). Electroactive behavior of graphene nanoplatelets loaded cellulose composite actuators. Compos. Part B Eng..

[96] Tang Z.H., Zhu W.B., Mao Y.Q., Zhu Z.C., Li Y.Q., Huang P., Fu S.Y. (2022). Multiresponsive Ti(3)C(2)T(x) MXene-Based Actuators Enabled by Dual-Mechanism Synergism for Soft Robotics. ACS Appl. Mater. Interfaces.

[97] Zhou J., Mulle M., Zhang Y., Xu X., Li E.Q., Han F., Thoroddsen S.T., Lubineau G. (2016). High-ampacity conductive polymer microfibers as fast response wearable heaters and electromechanical actuators. J. Mater. Chem. C.

[98] Kayser L.V., Lipomi D.J. (2019). Stretchable Conductive Polymers and Composites Based on PEDOT and PEDOT:PSS. Adv. Mater..

[99] Najathulla B.C., Kumar S., Deshpande A.S., Khandelwal M. (2023). PEDOT:PSS-bacterial cellulose bilayer actuators: From the movement of ions to deflection. Polym. Adv. Technol..

[100] Terasawa N., Asaka K. (2018). Self-standing cellulose nanofiber/poly(3,4-ethylenedioxythiophene):poly(4-styrenesulfonate)/ionic liquid actuators with superior performance. RSC Adv..

[101] Wang F., Wang L., Wu Z., Wang W. (2023). A low-voltage electro-ionic soft actuator based on graphene nanoplatelets-sulfonated cellulose nanowhisker combined with microfibrillated cellulose. J. Mater. Sci..

[102] Wang F., Jin Z., Zheng S., Li H., Cho S., Kim H.J., Kim S.-J., Choi E., Park J.-O., Park S. (2017). High-fidelity bioelectronic muscular actuator based on porous carboxylate bacterial cellulose membrane. Sens. Actuators B Chem..

[103] Reid L., Hamad W.Y. (2021). Electro-osmotic Actuators from Cellulose Nanocrystals and Nanocomposite Hydrogels. ACS Appl. Polym. Mater..

[104] Correia D.M., Lizundia E., Meira R.M., Rincón-Iglesias M., Lanceros-Méndez S. (2020). Cellulose Nanocrystal and Water-Soluble Cellulose Derivative Based Electromechanical Bending Actuators. Materials.

[105] Chen X., Ye Z., Yang F., Feng J., Li Z., Huang C., Ke Q., Yin Y. (2020). Magnetic cellulose microcrystals with tunable magneto-optical responses. Appl. Mater. Today.

[106] Tomás A.R., Gonçalves A.I., Paz E., Freitas P., Domingues R.M.A., Gomes M.E. (2019). Magneto-mechanical actuation of magnetic responsive fibrous scaffolds boosts tenogenesis of human adipose stem cells. Nanoscale.

[107] Kim J., Hyun J. (2021). Soft Magnetostrictive Actuator String with Cellulose Nanofiber Skin. ACS Appl. Mater. Interfaces.

[108] Ge W., Zhang F., Wang D., Wei Q., Li Q., Feng Z., Feng S., Xue X., Qing G., Liu Y. (2022). Highly Tough, Stretchable, and Solvent-Resistant Cellulose Nanocrystal Photonic Films for Mechanochromism and Actuator Properties. Small.

[109] Hartings M., Douglass K.O., Neice C., Ahmed Z. (2018). Humidity Responsive Photonic Sensor based on a Carboxymethyl Cellulose Mechanical Actuator. Sens. Actuators B Chem..

[110] Kuang Y., Chen C., Cheng J., Pastel G., Li T., Song J., Jiang F., Li Y., Zhang Y., Jang S.-H. (2019). Selectively aligned cellulose nanofibers towards high-performance soft actuators. Extrem. Mech. Lett..

[111] Yinan L., Jun W., Huixin L., Liulian H., Lihui C., Yonghao N., Qinghong Z. (2021). Highly transparent RCF/PTFE humidity and IR light dual-driven actuator with high force density, sensitivity and stability. Appl. Surf. Sci..

[112] Cao J., Zhou Z., Song Q., Chen K., Su G., Zhou T., Zheng Z., Lu C., Zhang X. (2020). Ultrarobust Ti(3)C(2)T(x) MXene-Based Soft Actuators via Bamboo-Inspired Mesoscale Assembly of Hybrid Nanostructures. ACS Nano.

[113] Jie W., Shuai J., Chao M., Jie G., Chunxia Y., Libin Z., Ziqiang S. (2022). Nacre-inspired composite film with mechanical robustness for highly efficient actuator powered by humidity gradients. Chem. Eng. J..

[114] Yang L., Cui J., Zhang L., Xu X., Chen X., Sun D. (2021). A Moisture-Driven Actuator Based on Polydopamine-Modified MXene/Bacterial Cellulose Nanofiber Composite Film. Adv. Funct. Mater..

[115] Li P., Su N., Wang Z., Qiu J. (2021). A Ti3C2Tx MXene-Based Energy-Harvesting Soft Actuator with Self-Powered Humidity Sensing and Real-Time Motion Tracking Capability. ACS Nano.

[116] Mohammadi M., Berggren M., Tybrandt K. (2023). Versatile Ultrasoft Electromagnetic Actuators with Integrated Strain-Sensing Cellulose Nanofibril Foams. Adv. Intell. Syst..

[117] Wei J., Jia S., Guan J., Ma C., Shao Z. (2021). Robust and Highly Sensitive Cellulose Nanofiber-Based Humidity Actuators. ACS Appl. Mater. Interfaces.

[118] Yang K., Cai W., Lan M., Ye Y., Tang Z., Guo Q., Weng M. (2022). Multi-responsive and programmable actuators made with nacre-inspired graphene oxide-bacterial cellulose film. Soft Matter.

[119] Wang F., Huang D., Li Q., Wu Y., Yan B., Wu Z., Park S. (2023). Highly electro-responsive ionic soft actuator based on graphene nanoplatelets-mediated functional carboxylated cellulose nanofibers. Compos. Sci. Technol..

[120] Cai G., Ciou J.H., Liu Y., Jiang Y., Lee P.S. (2019). Leaf-inspired multiresponsive MXene-based actuator for programmable smart devices. Sci. Adv..

[121] Rühlicke S., Zhang K. (2020). Synthesis of novel cellulose mixesters for transparent responsive films with switchable mechanical properties. Mater. Today Commun..

[122] Li Y., Wang J., Guo J., Fu C., Huang L., Chen L., Ni Y., Zheng Q. (2023). UV and IR dual light triggered cellulose-based invisible actuators with high sensitivity. Int. J. Biol. Macromol..

[123] Shu L., Zhang X.F., Wu Y., Wang Z., Yao J. (2023). Facile fabrication of strong and conductive cellulose hydrogels with wide temperature tolerance for flexible sensors. Int. J. Biol. Macromol..

[124] Chen M., Wan H., Hu Y., Zhao F., An X., Lu A. (2023). Rationally designed cellulose hydrogel for an ultrasensitive pressure sensor. Mater. Horiz..

[125] Yun T., Du J., Ji X., Tao Y., Cheng Y., Lv Y., Lu J., Wang H. (2023). Waterproof and ultrasensitive paper-based wearable strain/pressure sensor from carbon black/multilayer graphene/carboxymethyl cellulose composite. Carbohydr. Polym..

[126] Cui J., Li F., Wang Y., Zhang Q., Ma W., Huang C. (2020). Electrospun nanofiber membranes for wastewater treatment applications. Sep. Purif. Technol..

[127] Deng Y., Lu T., Cui J., Keshari Samal S., Xiong R., Huang C. (2021). Bio-based electrospun nanofiber as building blocks for a novel eco-friendly air filtration membrane: A review. Sep. Purif. Technol..

[128] Deng Y., Lu T., Zhang X., Zeng Z., Tao R., Qu Q., Zhang Y., Zhu M., Xiong R., Huang C. (2022). Multi-hierarchical nanofiber membrane with typical curved-ribbon structure fabricated by green electrospinning for efficient, breathable and sustainable air filtration. J. Membr. Sci..

[129] Cui J., Lu T., Li F., Wang Y., Lei J., Ma W., Zou Y., Huang C. (2021). Flexible and transparent composite nanofibre membrane that was fabricated via a “green” electrospinning method for efficient particulate matter 2.5 capture. J. Colloid Interface Sci..

[130] Lu T., Cao W., Liang H., Deng Y., Zhang Y., Zhu M., Ma W., Xiong R., Huang C. (2022). Blow-Spun Nanofibrous Membrane for Simultaneous Treatment of Emulsified Oil/Water Mixtures, Dyes, and Bacteria. Langmuir.

[131] Deng Y., Zhu M., Lu T., Fan Q., Ma W., Zhang X., Chen L., Min H., Xiong R., Huang C. (2023). Hierarchical fiber with granular-convex structure for highly efficient PM2.5 capture. Sep. Purif. Technol..

[132] Cui J., Wang Y., Lu T., Liu K., Huang C. (2021). High performance, environmentally friendly and sustainable nanofiber membrane filter for removal of particulate matter 1.0. J. Colloid Interface Sci..

[133] Ma W., Ding Y., Li Y., Gao S., Jiang Z., Cui J., Huang C., Fu G. (2021). Durable, self-healing superhydrophobic nanofibrous membrane with self-cleaning ability for highly-efficient oily wastewater purification. J. Membr. Sci..

[134] Deng Y., Lu T., Cui J., Ma W., Qu Q., Zhang X., Zhang Y., Zhu M., Xiong R., Huang C. (2022). Morphology engineering processed nanofibrous membranes with secondary structure for high-performance air filtration. Sep. Purif. Technol..

[135] Wang F., Huang D., Wu Y., Wang D. (2023). Ecofriendly low voltage high-performance ionic artificial muscles based on bacterial cellulose nanofibers reinforced with polyvinyl alcohol. J. Mater. Sci. Mater. Electron..

[136] Gu T., Bi H., Sun H., Tang J., Ren Z., Zhou X., Xu M. (2023). Design and development of 4D-printed cellulose nanofibers reinforced shape memory polymer composites: Application for self-deforming plant bionic soft grippers. Addit. Manuf..

[137] Wang X., Tang S., Wu Z., Fang J., Qin X., Wei L. (2021). Research status of biomass-based composite films with high barrier properties. J. For. Eng..

[138] Mao F., Long L., Zeng G., Chen H., Li Y., Zhou W. (2022). Achieving excellent electromagnetic wave absorption property by constructing VO2 coated biomass carbon heterostructures. Diam. Relat. Mater..

[139] Xu J., Zhai Q., Long F., Jiang X., Han S., Jiang J. (2022). Electrocatalytic oxidation and reduction of biomass-derived chemicals: A review. J. For. Eng..

[140] Deng W.-N., Li Y.-H., Xu D.-F., Zhou W., Xiang K.-X., Chen H. (2022). Three-dimensional hierarchically porous nitrogen-doped carbon from water hyacinth as selenium host for high-performance lithium–selenium batteries. Rare Met..

[141] Guo Z., Han X., Zhang C., He S., Liu K., Hu J., Yang W., Jian S., Jiang S., Duan G. (2023). Activation of biomass-derived porous carbon for supercapacitors: A review. Chin. Chem. Lett..

